# Gene expression variability in long-term survivors of childhood cancer and cancer-free controls in response to ionizing irradiation

**DOI:** 10.1186/s10020-023-00629-2

**Published:** 2023-03-30

**Authors:** Caine Lucas Grandt, Lara Kim Brackmann, Ronja Foraita, Heike Schwarz, Willempje Hummel-Bartenschlager, Thomas Hankeln, Christiane Kraemer, Sebastian Zahnreich, Philipp Drees, Johanna Mirsch, Claudia Spix, Maria Blettner, Heinz Schmidberger, Harald Binder, Moritz Hess, Danuta Galetzka, Federico Marini, Alicia Poplawski, Manuela Marron

**Affiliations:** 1 Leibniz Institute for Prevention Research and Epidemiology-BIPS, Achterstr. 30, 28359 Bremen, Germany; 2 Faculty of Human and Health Sciences, University of Bremen, Bremen, Germany; 3 Institute of Organismic and Molecular Evolution, Molecular Genetics and Genome Analysis, Johannes Gutenberg University Mainz, Mainz, Germany; 4 Department of Radiation Oncology and Radiation Therapy, University Medical Center of the Johannes Gutenberg University Mainz, Mainz, Germany; 5 Department of Orthopaedics and Traumatology, University Medical Center of the Johannes Gutenberg University Mainz, Mainz, Germany; 6 Radiation Biology and DNA Repair, Technical University of Darmstadt, Darmstadt, Germany; 7 Division of Childhood Cancer Epidemiology, German Childhood Cancer Registry, Institute of Medical Biostatistics, Epidemiology and Informatics (IMBEI), University Medical Center of the Johannes Gutenberg University Mainz, Mainz, Germany; 8 Institute of Medical Biostatistics, Epidemiology and Informatics (IMBEI), Center of the Johannes, University Medical, Gutenberg University, Mainz, Germany; 9 Institute of Medical Biometry and Statistics, University Medical Center, Freiburg, Germany

**Keywords:** NGS, RNA-Seq, Childhood cancer, Radiation response, High dose, Low dose, Pan-cancer, Kikme study

## Abstract

**Background:**

Differential expression analysis is usually adjusted for variation. However, most studies that examined the expression variability (EV) have used computations affected by low expression levels and did not examine healthy tissue. This study aims to calculate and characterize an unbiased EV in primary fibroblasts of childhood cancer survivors and cancer-free controls (N0) in response to ionizing radiation.

**Methods:**

Human skin fibroblasts of 52 donors with a first primary neoplasm in childhood (N1), 52 donors with at least one second primary neoplasm (N2 +), as well as 52 N0 were obtained from the KiKme case–control study and exposed to a high (2 Gray) and a low dose (0.05 Gray) of X-rays and sham- irradiation (0 Gray). Genes were then classified as hypo-, non-, or hyper-variable per donor group and radiation treatment, and then examined for over-represented functional signatures.

**Results:**

We found 22 genes with considerable EV differences between donor groups, of which 11 genes were associated with response to ionizing radiation, stress, and DNA repair. The largest number of genes exclusive to one donor group and variability classification combination were all detected in N0: hypo-variable genes after 0 Gray (n = 49), 0.05 Gray (n = 41), and 2 Gray (n = 38), as well as hyper-variable genes after any dose (n = 43). While after 2 Gray *positive regulation of cell cycle* was hypo-variable in N0, (*regulation of*) *fibroblast proliferation* was over-represented in hyper-variable genes of N1 and N2+. In N2+, 30 genes were uniquely classified as hyper-variable after the low dose and were associated with the *ERK1/ERK2 *cascade. For N1, no exclusive gene sets with functions related to the radiation response were detected in our data.

**Conclusion:**

N2+ showed high degrees of variability in pathways for the cell fate decision after genotoxic insults that may lead to the transfer and multiplication of DNA-damage via proliferation, where apoptosis and removal of the damaged genome would have been appropriate. Such a deficiency could potentially lead to a higher vulnerability towards side effects of exposure to high doses of ionizing radiation, but following low-dose applications employed in diagnostics, as well.

**Supplementary Information:**

The online version contains supplementary material available at 10.1186/s10020-023-00629-2.

## Introduction

Application of high-dose ionizing radiation (HDIR) in radiotherapy can cause acute (e.g., inflammation) or late adverse reactions, such as the development of a second primary neoplasm (Tukenova et al. 2011; Spector et al. 2015; Inskip et al. 2016; Scholz-Kreisel et al. 2018), the risk for which is additionally increased if exposure to HDIR occurs at a young age (Hodgson et al. 2017). However, it is still unclear why only a fraction of childhood cancer survivors, regardless of therapy, develops second primary neoplasms later in life (Kutanzi et al. 2016). An explanation may be provided by the high degree of individual biological variability in pathways like radiation-response, which are needed in reaction to stressors such as radiotherapy (Smirnov et al. 2012; Hornhardt et al. 2014; Borràs-Fresneda et al. 2016; Seibold et al. 2019). Individual genetic variations such as single nucleotide polymorphisms and copy number alterations affect the expression variability (EV) (Li et al. 2010), which subsequently modulates the translation into cellular function. Moreover, non-sequence-based genetic factors like epigenetic modifications (e.g., methylation) also impact gene expression magnitude and EV while posing as a link between environment, lifestyle, and genome (A I et al. 2013; Bashkeel et al. 2019). After several decades of differential expression analyses and testing for differences in mean expression, the variability of an expressed gene may additionally impact phenotypes, modulate fitness, be indicative for disease (Ho et al. 2008; Li et al. 2010; Mar et al. 2011; Corrada Bravo et al. 2012; Alemu et al. 2014), and underlie evolutionary selection (Feinberg and Irizarry 2010; Zeller et al. 2010; Bashkeel 2019). To date, only a few studies focused on more elaborate methodologies to estimate the EV (Oleksiak et al. 2002; Storey et al. 2007; Ho et al. 2008; Li et al. 2010; Stranger et al. 2012; Breschi et al. 2016). However, some of these approaches perform poorly when estimating the EV of lowly expressed genes (Alemu et al. 2014; Simonovsky et al. 2019). To address this issue, the study group of Alemu and colleagues was the first to define the term ‘EV’, establishing it as a measurement that separates expression variability from overall expression levels (Alemu et al. 2014) by defining EV as the ratio of variance_observed_ to variance_expected_. As their approach was still sensitive to outliers and moderately biased towards lowly expressed genes (Simonovsky et al. 2019), others thus modified it by using the median absolute deviation (MAD) instead of the standard deviation (SD) to add robustness against outliers (Wu et al. 2014; Bashkeel et al. 2019). Bashkeel et al. (2019) additionally filtered for bimodally expressed genes and used bootstrapping to compute the ‘observed MAD’. Similar to Corrada et al. (2012), genes were then classified based on a defined range of the EV-metric and classifications were then cross-validated to reduce sampling error and to provide further robustness. In cancer, the EV bears information concerning oncogenesis (Afsari et al. 2014) and methylation-mediated changes in EV can explain the heterogeneity between tumour subtypes (Hansen et al. 2011; Landau et al. 2014; Ecker et al. 2015), as well as adverse clinical outcomes (Landau et al. 2014; Yard et al. 2016). Corrada et al. (2012) showed that an increased EV of specific genes enabled machine-learning-based distinction between healthy and tumour tissue samples. To this day, no study has examined the EV in healthy tissue from subjects with a history of childhood cancer. However, the information on EV might explain why some long-term survivors of childhood cancer develop further primary neoplasms and some do not. This work, therefore, aims to identify the expression variability in primary skin fibroblasts from long-term survivors of childhood cancer without (N1) and with at least one second primary malignancy (N2+), as well as from cancer-free controls (N0), after exposure to a high (2 Gray, HDIR) or low (0.05 Gray, LDIR) dose of ionizing radiation. To do so, gene expression data, previously examined for differential expression (Grandt et al. 2022) were examined to identify patterns in EV potentially related to the participants’ onset of first and second primary neoplasms, adapting the pipeline proposed by Bashkeel et al. (2019) and furthermore to explore whether there are variability patterns that might be used to differentiate N1 and N2+ through application of machine learning algorithms.

## Methods

### Study design, participants, and differential gene expression

The KiKme nested case–control study was conducted to identify genetic predispositions associated with paediatric cancer and second primary neoplasms, potentially initialised by exposure to ionizing radiation during radiation therapy for the first cancer in childhood or radiation diagnostics prior to the first childhood cancer. For this purpose, biosamples, as well as data on lifestyle, medical history, and history of radiation exposure were collected. The KiKme study design (Marron et al. 2021), a detailed description of the establishment of the radiation experiments (Brackmann et al. 2020), the differential expression analysis, as well as a description of the study sample used in this work (Grandt et al. 2022), can be found in detail elsewhere. In short, the median age of donors with at least one second primary neoplasm was 32.0 years, and 32.5 years of donors with only a first neoplasm in childhood, respectively (interquartile range 28.0–38.2 years). The median age of cancer-free controls was 33.0 years (interquartile range 27.8–38.0) at the time of sampling. Half (51.9%) of the participants were female. All subjects included in this study were matched by age at recruitment and sex. The long-term survivors of childhood cancer were additionally matched by first cancer site, as well as age at and year of the first cancer diagnosis. Primary skin fibroblasts were sampled from skin biopsies of 156 donors with cancer in childhood without a second primary neoplasm (N1, n = 52), donors with cancer in childhood and at least one second primary neoplasm (N2 + , n = 52), and cancer-free controls (N0, n = 52).

### RNA-sequencing and processing

In short, fibroblasts were cultured for ~ 14 days, then irradiated as triplets with fibroblasts of the matched donors with 0, 0.05 (LDIR), or 2 (HDIR) Gray, respectively. RNA was then isolated 4 h after exposure. Samples with an RNA integrity number < 7 were not used for the subsequent library preparation. The libraries were processed on a HiSeq2500 instrument (Illumina, San Diego, California, USA) which was set to high-output mode (Nucleic Acids Core Facility, Faculty of Biology, Univ. Mainz). The reads were then generated using the *TruSeq Single Read Cluster Kit v3* and the *TruSeq SBS Kit v3* (Illumina, San Diego, California, USA). Here, single-end reads had a length of 51 base pairs using single indices (8). The base calling was performed by *Real-Time Analysis* (Version 1.8.4) and the resulting data were then converted into FASTQ format using *bcl2fastq* (Version 1.8.4, Illumina, San Diego, California, USA). The raw reads were separated from the adapter sequences using *Trimmomatic* (Bolger et al. 2014) and the processed reads were aligned to the human reference genome (GRCh38) using *STAR* (Dobin et al. 2013). The expression per gene was then computed as the number of aligned reads per gene, quantified using *FeatureCounts* (Liao et al. 2014). The data were then normalized using the *voom* method (Law et al. 2014) for the detection of differentially expressed genes (adjusted for age at recruitment and sex) via *limma*, (Ritchie et al. 2015) (Additional file 1a) and *DESeq2* (Love et al. 2014) for the EV pipeline.

### Computation of expression variability and classification of genes

We used a modified version of the pipeline by Bashkeel et al. (2019) for computing the EV and the subsequent classification into hypo-, non-, and hyper-variable genes. As with that pipeline, this work also solely examined the EV of genes showing a unimodal distribution across all three donor groups per experiment. Hereby the assumption was that a high EV represented the widened ranges of count values across the median. Genes where the expression showed multimodal patterns, thus having more than one discrete state (e.g., differential expression between the phenotypes) were examined in another work using differential expression analysis (Grandt et al. 2022). Contrary to Bashkeel et al., (2019) who used microarray data and thus computed bimodal genes using Gaussian distribution, we used SIBERG (version 2.0.3) to calculate a bimodal index for RNA-Sequencing data (Wang et al. 2009) with default settings (zeroPercentThr = 0.2, base = exp(1) and eps = 10). To account for the normalization of mRNA data, a vector, containing the respective normalization factors for each sample calculated with DESeq2 (Love et al. 2014), was provided to the SIBERG algorithm. Raw RNA-sequencing counts of bimodal expression distributions were analysed for all donor groups (N2 + , N1, and N0) using a log-normal mixture model (Tong et al. 2013). Moreover, as the same genes may show different expression patterns depending on the radiation dose, data of each experiment (sham irradiation, LDIR, and HDIR) were analysed separately. Thus, genes with a bimodal distribution, identified by a bimodal index >  = 1.1, were removed from the data for further analyses (Additional file 1b) (Tong et al. 2013). Assuming that the EV and the participants’ cancer history could be associated, the following computational steps were applied to the data stratified by donor group and radiation dose. First, the MAD_oberserved_ was calculated for each gene per donor group and radiation treatment as the median of 1,000 bootstrap iterations. Second, the MAD_predicted_ was estimated by fitting a non-parametric local polynomial regression (loess) function. The EV for each gene was then calculated as the difference MAD_oberserved_—MAD_predicted_. Genes were classified as hypo-, non, or hyper-variable if their EV was below, within, or above the interval: Median_EV_ ± 3 $\cdot$ MAD_EV_. Here, x̃_EV_ is the median EV in the N0 group. The MAD_EV_ was again computed as the median of 1000 bootstrap iterations. Assuming that the EV of the N0 group is associated with the non-cancer phenotype, EV in the N2 + and N1 groups were not classified by their internally defined ranges, but by the range defined through expressional data of the N0 group. The resulting classifications were then cross-validated for each treatment by randomly splitting each donor group (N0, N1, N2 +) in half and repeating the complete classification pipeline 10 times. Next, classification of genes was confirmed using a binomial test, where the success was defined as a concordant classification in the first (split 1) and in the second (split 2) half of each donor group, as well as in the whole dataset. Here, the alternative hypothesis was defined as the true probability of success being greater than 0.5. The resulting *p*-values from the binomial tests were then adjusted for false discovery at a rate of 0.05. The resulting data sets (Additional file 3) were then analysed for overlaps and displayed using upsetR (Conway et al. 2017). Genes whose classification as hypo- or hyper-variable was verified by cross-validation and that were part of unique gene sets after the set-based analysis for overlap with *upsetR* (e.g., genes that were only classified as hypo-variable in N0 after LDIR) were then subjected to the Gene Ontology (GO) over-representation analysis (see below).

### Sensitivity analyses

This workflow was also applied to subsets of the data after stratifying by sex. Moreover, to examine the EV without potential confounding introduced by tobacco smoking or alcohol consumption, we repeated the analysis pipeline after excluding donor triplets where at least one donor (i) had smoked tobacco for over ten pack years (23 of 52 triplets remaining) and/or (ii) consumed more than 2 alcoholic beverages per day (38 of 52 triplets remaining). Due to an otherwise too strongly reduced sample size, we only filtered for the values representing the questionnaire categories for the highest consumption of tobacco smoking and alcohol, respectively. For all sensitivity analyses, we applied an appropriate higher threshold of 1.3 for the bimodal index due to the reduced sample sizes (n_Female_: 81, n_Male_: 75, as well as n_healthy_: 60) as recommended by Tong et al. (2013).

### Comparison of methods to compute variability estimates

To evaluate the performance of EV as a measurement of gene expression variability, we also calculated the coefficient of variation (CV = SD/mean) and the standardized MAD (d = MAD/median) for each gene per dose and donor group. We then compared the influence of expression magnitude on all three metrics using Kendall’s Tau. This is a measure robust to departure from linear associations, to assess the correlation between the variability metric and the median expression level.

### Construction of the candidate gene list

To reduce the high complexity of our data, we also constructed a candidate list, containing genes assumed to be associated with the radiation response. To do so, we collected genes curated in the RadAtlas (Xu et al. 2020), genes known to be involved in DNA repair (Knijnenburg et al. 2018), as well as genes annotated to the GO terms response to stress and response to radiation (The Gene Ontology Consortium 2018), and the top 100 genes with regard to *p*-value from the differential gene expression analysis in reaction to LDIR and HDIR we have previously conducted (Grandt et al. 2022). We then filtered the genes in our data that were classified as hypo- and hyper-variable in any combination of donor group and radiation dose for genes on the candidate list. Furthermore, we acquired information in the literature on genes whose methylation may be potentially affected by radiation and examined these regarding their respective EV in reaction to the different radiation doses (Antwih et al. 2013; Miousse et al. 2017).

### Gene Ontology over-representation analysis

Genes whose classification as hypo- or hyper-variable confirmed by cross-validation were additionally analysed for over-representation using the ConsensusPathDB (Kamburov et al. 2012). According to best practice for pathway/functional analyses (e.g., the over-representation analysis used in this work), a gene set is compared to a given list of total genes measured in an experiment, called background (Wijesooriya et al. 2022). As such, gene lists per radiation dose after exclusion of bimodally expressed genes were used (Additional file 3). Resulting GO terms of the category biological process were filtered for adjusted *p*-values < 0.05 (Benjamini–Hochberg procedure (Benjamini and Hochberg 1995)). This was done for (i) each combination of radiation dose, EV-classification, and donor group, as well as (ii) unique gene sets identified in the upsetR-analysis. The complete list of GO terms for (i) can be found in Additional file 4. These GO term results for each radiation dose were also examined for overlap between the donor groups per radiation dose and EV classification and filtered for the top 5 GO terms regarding the adjusted *p-*value. For (ii), respective GO terms were summarized into clusters using *REVIGO* (Supek et al. 2011) with an allowed semantic similarity of GO terms set to 0.7 and the database set to *Homo sapiens*. The results were then extracted and plotted as tree maps using the R script provided by the platform. In these, tile sizes of the tree maps were defined to represent the adjusted *p-*value of each respective GO term (Additional file 6).

### Application of classification algorithms

We further used selected gene sets uniquely classified as hyper-variable in reaction to LDIR in N2 + to distinguish between N1 and N2 + , using N1 as the reference class. For this purpose, *MLSEQ* (Goksuluk et al. 2019) was employed; an R package designed to apply a plethora of classifiers to RNASeq data (Additional file 2d). The models were trained on a randomly assigned 20% training split of the data and their respective accuracy was then examined using the remaining 80% of the data. The models were trained with the “validation method” set to 10 times repeated fivefold cross-validation and the tune length (number of values used for the tuning parameter, if the respective classifier had a tuning parameter) set to 10, according to the readme of the package. In sparse models (e.g., voomNSC) that set parameters of the model to 0 if not relevant for the outcome prediction accuracy, the selectedGenes-function was used to identify potential biomarker genes that contributed most to the discrimination function of the classifier.

## Results

### Bimodal genes, computation of expression variability, and gene classification

**Fig. 1:**
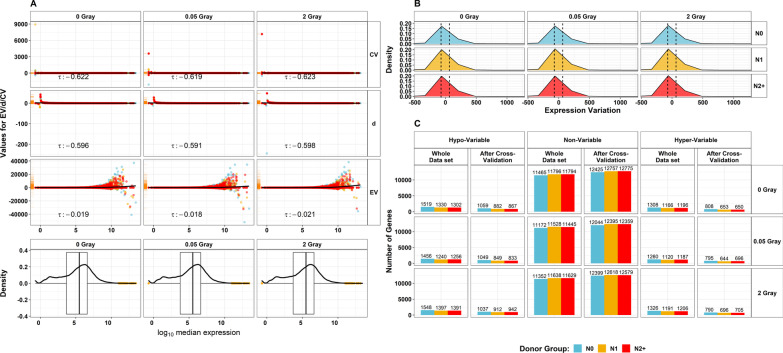
Comparison of methods for quantification of expression variability and overview of distribution and subsequent variability classification: **A** Comparison of different metrics that are typically used for the characterization of expressional variability. Scatter plots showing the association of the coefficient of variation (CV, standard deviation/mean), standardized median absolute deviation (d, median absolute deviation (MAD)/median), and expression variability (EV, computed as MAD_observed_-MAD_expected_) on the respective y-axes with the median expression on the x-axis. Kendall’s correlation coefficient (τ) for the correlation of each variability metric with the median gene expression is provided per facet. The yellow stripes on the left show the value distribution of each variability metric. The colours of the dots represent the donor groups (N0 = fibroblasts of cancer-free controls, N1 = fibroblasts of childhood cancer survivors without a second primary neoplasm, N2 +  = fibroblasts of childhood cancer survivors with at least one second primary neoplasm) Below, the distribution of the median gene expression is presented as box- and density plots. **B** Density plots showing the distribution of the EV (truncated using the smallest Q_01_ (N0 after LDIR) EV = − 427.9 to the largest Q_99_ (N0 after 2 Gray) EV = 1204.8 for reasons of legibility), also stratified by donor group, and radiation dose. Dashed lines imply the ranges employed for the classification into hypo-, non-, and hyper-variable, whereas genes with an EV lower than the left dashed lines were classified as hypo-variable and genes with an EV higher than the right dashed line were conversely classified as hyper-variable. Genes with an EV between the dashed lines were classified as non-variable. **C** Bar charts showing the number of genes that were classified as hypo-, non-, and hyper-variable, using the whole data set and the number of genes with stable classification confirmed by cross-validation. All data were stratified by exposed radiation dose (0, 0.05, and 2 Gray)

The data contained expression information for 14,756 genes. Of these, 97 genes (0.66%) in the sham irradiation data set, 511 genes (3.46%) in the LDIR data set, and 164 genes (1.11%) in the HDIR data set had a bimodal index higher or equal to 1.1 and were excluded before computation of the EV (Additional file 1a). The comparison of EV, the CV, and the standardized MAD (d) with the median expression showed negative correlation values which were more pronounced for CV and d (tau_CV_ = − 0.622/− 0.619/− 0.623, tau_d_ = − 0.596/− 0.591/− 0.598 for 0, 0.05, and 2 Gy, respectively) than for EV (tau_EV_ = − 0.019/− 0.018/− 0.021 for 0, 0.05, and 2 Gy, respectively), whereas the EV was more dispersed in very high expression levels. The distribution of EV-values was similar across donor groups and radiation doses (Fig. 1b). The number of hypo- and hyper-variable genes decreased in all data sets after cross-validation (Fig. 1c). After all radiation doses, most genes were classified as hypo- (sham irradiation: 1059, LDIR: 1049, HDIR: 1037) and hyper- (sham irradiation: 808, LDIR: 795, HDIR:790) variable in N0, while there were about 20% fewer genes classified as hypo- or hyper-variable in N1 and N2 +. Comparing gene classifications, we identified 461 hypo- and 333 hyper-variable genes that had identical classifications in fibroblasts of all donors at any radiation dose (Additional files 2 and 5a). The following 4 intersect groups consisted exclusively of N0-data sets. These were hypo-variable genes after sham irradiation (n = 49 genes), LDIR (n = 41 genes), and HDIR (n = 38 genes). Additionally, 43 genes were only hyper-variable in N0 after any radiation dose. Of these 171 genes in total, 49 (sham irradiation: *BAG5, CCND1, DNAJB2, EIF2AK2, FH, GNAQ, LAMB2, OSMR, POLR2C, PPIG, PSMA3, PSMB3, TOLLIP, VDAC3*, and *WDR48*; LDIR: *ABCF3*, *ARID1A*, *CCM2*, *CIRBP*, *GTF2H1*, *PPP1R10*, *PRDX5*, *PSMD4*, *PSMD5*, *RPS19*, *SIGMAR1*, *SRPK2*, and *STK25;* HDIR: *LASP1*, *CST3*, *GLB1*, *MSH6*, *RAD21*, *RAD23A*, *SIN3A*, *TXNRD1*, *ULK1*, and *YBX3;* Hyper-variable after all doses: *ATF4*, *CAMK1D*, *DKK2*, *FBLN5*, *IL1R1*, *KLF2*, *LOXL3*, *MSRB3*, *RCN3*, *SREBF2*, and *TRAIL*) were also present in our candidate list, which consisted originally out of 4,810 genes.

### Sensitivity analyses

#### Bimodal genes and differential gene expression

To examine whether genes were bimodally expressed e.g., because they were differentially expressed between phenotypes, we compared the excluded bimodally expressed genes (Additional file 1a) with their respective data on differential expression status between donor groups (Additional file 1b). After exposure to the sham-irradiation (n = 97) and HDIR (n = 164), half of the identified bimodally expressed genes were also differentially expressed comparing N1 and N2 + with N0 (Additional file 7a). After LDIR, about one-third of the 511 bimodal genes were also differentially expressed genes comparing donor groups (Additional file 7b). Genes showing the highest values for the bimodal index (≥ 2.5) were mostly genes that were not differentially expressed between donor groups (Additional file 7a).

#### Sex

To examine differences in EV we stratified the data by sex and repeated the analysis pipeline. After sham-irradiation, 281 genes from the female and 35 from the male data set were excluded, compared to 97 genes in the combined data (Additional file 8a). In the LDIR data set, 138 genes from the female and 1553 from the male data were excluded (511 genes in the complete data set). In the HDIR data, 68 genes from the female and 151 genes from the male data had a bimodal index over 1.3 and were excluded (complete data: 164). The values for the EV were comparable between males and females (adjusted r^2^: 0.779; Kendall’s tau: 0.786; Additional file 8b). The number of genes per classification was comparable between sexes (Additional File 8c), and the overlap of hypo- and hyper-variable genes, stratified by radiation dose, sex, and donor group was persistent along the classification as hyper- (n = 531) and hypo- (n = 400) variable (Additional file 8d).

#### Exclusion of smokers and participants with heavy alcohol consumption

After filtering out donor triplets containing participants with strong smoking and drinking behaviour, as well as individuals with missing values on these variables, 20 triplets remained for this sensitivity analysis. Thus, after the exclusion of donor triplets with missing values, participants without any heavy smokers or drinkers in their respective triplets summed to a total of 60, and donors from triplets with at least one heavy drinker or smoker added up to 54 persons. The number of bimodally expressed genes was much higher here than in the analysis with all participants (n_SI_: 1280, n_LDIR_: 1726, and n_HDIR_: 527; Additional file 8e). Subsequently, the number of genes classified as hypo-/hyper-variable was lower than in the whole data set (Additional file 8f). Computed EV values from donor triplets termed *healthy*, meaning not heavy drinker, nor heavy smoker and unhealthy based on smoking and alcohol consumption were different (adjusted r^2^: 0.377; Kendall’s tau: 0.683, Additional file 8g). A comparison of data from triplets without heavy smoking and alcohol consumption showed the largest overlaps across all hyper- and all hypo-variable sets of genes, respectively (Additional file 8h). We further examined whether the number of bimodally expressed genes was explained by the exclusion of participants termed unhealthy based on smoking and alcohol consumption. Thus, we randomly generated samples with comparable large (n = 96) and small (n = 60) subsamples. These showed the same fluctuation in the number of bimodally expressed genes irrespective of metadata, solely explained by sample size (Additional file 8i).

### Candidate genes and methylation status

**Fig. 2:**
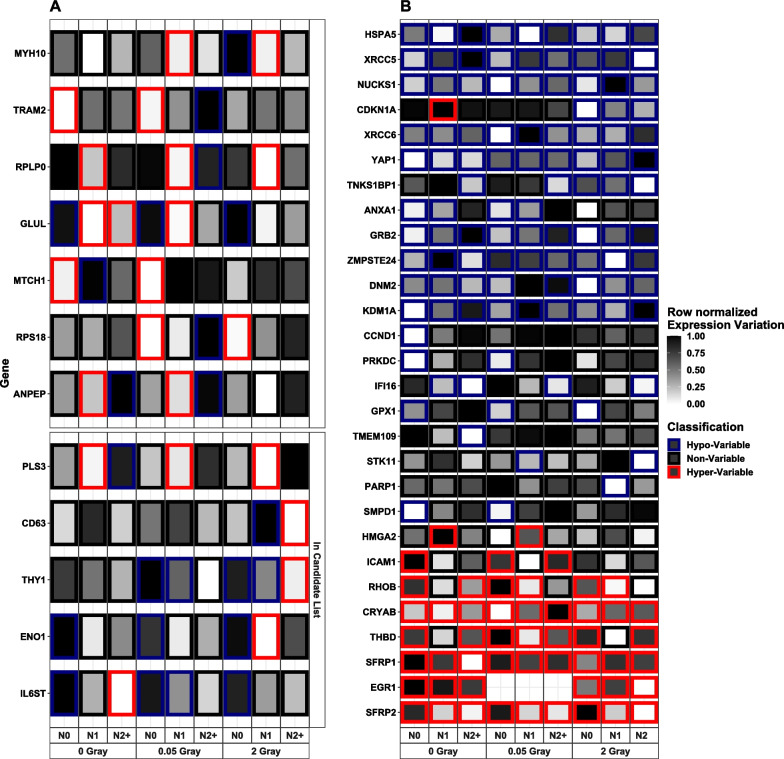
Heat maps of genes with functional interest: which were **A** classified both as hyper- and hypo-variable in any data set and **B** associated with the GO term response to radiation and -showed a classification range from to hyper-variable across all donor groups. Genes in **A** were additionally filtered for genes found in the RadAtlas database for radiation-associated genes, genes associated with DNA repair functions, or that were annotated to the Gene Ontology (GO) terms response to stress or response to radiation (see facet Candidate List). N0 = fibroblasts of cancer-free controls, N1 = fibroblasts of childhood cancer survivors without a second primary neoplasm, and N2 +  = fibroblasts of childhood cancer survivors with at least one second primary neoplasm

Based on our literature research, we identified 59 genes that would potentially show radiation-dependent methylation patterns. Of these, 50 genes were present in our data and 35 of these 50 genes were classified as non-variable in all combinations of donor group and radiation doses. The remaining 15 genes are depicted in Additional file 9. Here, *YWHAQ*, *YWHAG*, *YWHAE*, *YWHAB*, as well as *RAD23B* were classified as hypo-; *CDH13* and *IGFBP3* as hyper-variable in all donor group- and radiation dose-combinations. *CCND1* had a heterogeneous EV at sham-irradiation that aligned at LDIR and HDIR across all donor groups. *CDKN1A* was non-variable in all donor groups after sham irradiation and hypo-variable in all donor groups after HDIR. *ASPH* was hypo-variable in N0 after LDIR and in N0 and N1 after HDIR. The EV of *IGF1R* was dispersed across donor groups, whereas its expression was hyper-variable after sham-irradiation in N1, after LDIR in N0 and N2 + , and after HDIR in N1 and N2 + . Comparing the classification of genes between different donor groups and radiation doses, we identified 22 genes (*ALDOA*, *ANPEP*, *CCNG1*, *CD63*, *CDKN1A*, *ENO1*, *GLUL*, *IL6ST*, *IMPDH2*, *LIMS1*, *LRP1*, *MTCH1, MXRA8*, *MYH10, PLS3*, *RPLP0*, *RPS18*, *RPS27L*, *SPTBN1*, *THY1*, *TMEM119*, and *TRAM2*) that showed a large enough difference in EV to be classified as hypo-variable in any donor group and hyper-variable in at least one of the other donor groups (Fig. 2a). Of these genes, 11 (*CCNG1*, *CD63*, *CDKN1A*, *ENO1*, *GLUL*, *IL6ST*, *LRP1*, *MYH10*, *PLS3*, *RPS27L*, and *THY1*) were also present in our candidate list.

### Gene Ontology analysis

**Fig. 3:**
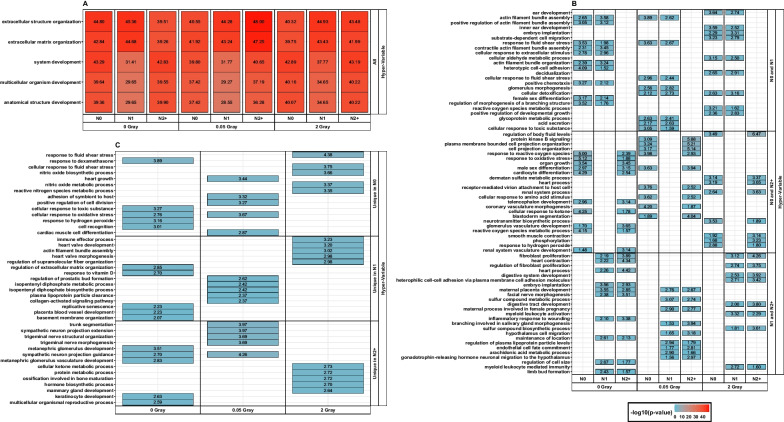
Summary of the top 5 Gene Ontology terms regarding the adjusted *p*-value: stratified by group-wise overlap (Top 5 in **A**) all groups, **B** overlap of two donor groups, and **C** unique in one group), per radiation dose for genes classified as hypo-variable. Inside the tiles, each respective adjusted -log_10_ (*p*-value) is provided. Since a *p*-value was computed per group for **A** and **B**, the top 5 GO terms e.g., found only in N0 and N1, but not in N2 + post-HDIR might differ. In this case, the top 5 for N0 and N1 are shown each, resulting in more than 5 terms. N0 = fibroblasts of cancer-free controls, N1 = fibroblasts of long-term survivors of childhood cancer without a second primary neoplasm, N2 +  = fibroblasts of long-term survivors of childhood cancer with at least one second primary neoplasm

**Fig. 4:**
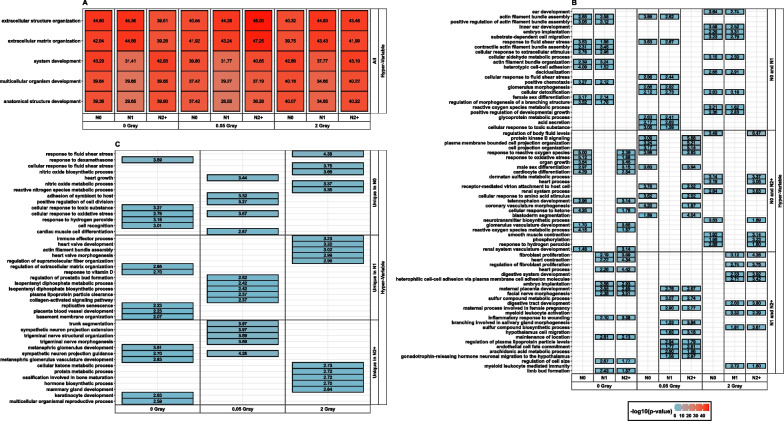
Summary of the top 5 Gene Ontology terms regarding the adjusted *p*-value: stratified by group-wise overlap (Top 5 in **A** all groups, **B** overlap of two donor groups, and **C** unique in one group), per radiation dose for genes classified as hyper-variable. Inside the tiles, each respective adjusted − log_10_ (*p*-value) is provided. Since a *p*-value was computed per group for **A**) and **B**), the top 5 GO terms e.g., found only in N0 and N1, but not in N2 + post-HDIR might differ. In this case, the top 5 for N0 and N1 are shown each, resulting in more than 5 terms. N0 = fibroblasts of cancer-free controls, N1 = fibroblasts of long-term survivors of childhood cancer without a second primary neoplasm, N2 +  = fibroblasts of long-term survivors of childhood cancer with at least one second primary neoplasm

**Fig. 5:**
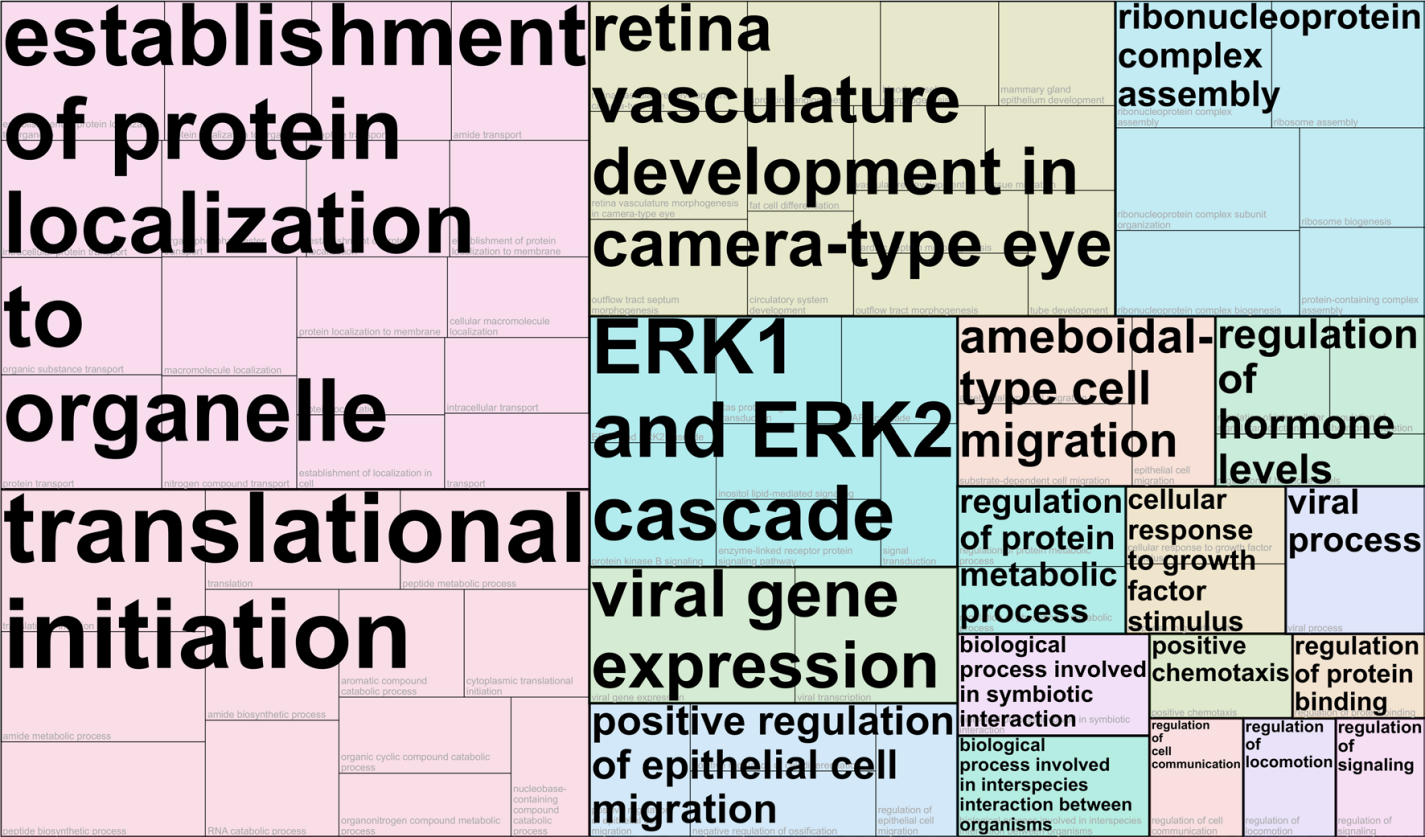
Tree map summarizing over-represented Gene Ontology terms for genes that were only classified as hyper-variable (n = 30) in fibroblasts of long-term survivors of childhood-cancer with at least one second primary neoplasm in reaction to exposure to 0.05 Gray

A comparison of GO term results showed a term consistency across variability-classifications (Additional file 5). 385 terms were over-represented in hyper-, and 276 GO terms were over-represented for hypo-variable genes of all donor groups across all radiation doses. Additional 76 GO terms were over-represented after all radiation doses for both, hypo- and hyper-variable genes. The GO term cellular response to radiation was associated with hypo-variable genes of all donor groups after all radiation treatments (Additional file 4). Its child-term cellular response to ionizing radiation was associated with the hypo-variable genes of N1 after HDIR and in N2 + data after all radiation doses, including the sham irradiation. Corresponding hypo- and hyper-variable genes are shown in Fig. 2b. Among the top 5 GO terms over-represented in hypo-variable gene sets of all donor groups, protein/macromolecule localization and intracellular transport were present after all radiation doses (Fig. 3).Prominent GO terms that were over-represented for hyper-variable gene sets of all donor groups after all radiation doses were extracellular structure/matrix organization, system development, multicellular organism development, and anatomical structure development (Fig. 4)*.* Next, we analysed the unique hypo-variable genes for N0 (Additional file 3b). After sham irradiation, these genes were clustered as protein modification process (Additional file 6a). In reaction to LDIR, the GO terms for the uniquely hypo-variable genes of N0 were transcription initiation-coupled chromatin remodelling, regulation of hematopoietic progenitor cell differentiation, RNA catabolic process, and negative regulation of hydrolase activity (Additional file 6b). After HDIR, clusters were negative regulation of catalytic activity and regulation of cellular process (Additional file 6c) Besides the unique hypo-variable genes, 43 genes were hyper-variable only in N0 across all radiation doses. These were functionally clustered into lung development, cellular response to laminar fluid shear stress, and neutrophil migration, Following the unique genes for N0, the next largest set of genes was among the hypo-variable genes in N2 + after HDIR (n = 30). The associated GO term was N-glycan processing (Additional file 6e). Additionally, there were 30 genes uniquely classified as the hyper-variable genes for N2 + after LDIR (n = 30). Among this identified set of genes, the associated GO terms were clustered into establishment of protein localization to organelle, translational initiation,* ERK1 *and* ERK2 *cascade, among others containing *ERK1 *and* ERK2 *cascade (*RRAS, ANGPT1, TNFAIP8L3, NRP1*), protein kinase B signaling (*RRAS, FGF5, ANGPT1, TNFAIP8L3*), *MAPK cascade* (*TNFAIP8L3, ROBO1, RRAS, FGF5, ANGPT1, NRP1*), Ras protein signal transduction [(*ROBO1, RRAS, RALGPS2, NRP1*), Fig. 5].We additionally filtered the data for the expression values for these 30 genes of N1 and N2 + post-LDIR and applied several classification algorithms (Additional file 2d). The voomNSC algorithm performed best with an accuracy of 0.68, sensitivity of 65.71% and a specificity of 70.27% using on average 14 of the 30 genes for the classification (*ANGPT1, CCDC71L, EIF3F, FGF5, IER3, KCNK15, NRP1, RGMB, ROBO1, RPL11, RPL13A, RPL3, RPS24, SETBP1*). GO terms associated with these genes were amongst others subsumed under the terms translational initiation and cellular response to growth factor stimulus [also containing *MAPK cascade*, (Additional file 6f)].

## Discussion

In this study, we adapted a pipeline for the computation of expression variability of microarray expression data to next-generation RNA-sequencing data to compare the EV in primary fibroblasts of a collective of cancer-free controls and childhood cancer survivors with and without at least one second primary neoplasm after treatment with different doses of ionizing radiation. This method was less affected by low expression values than metrics usually employed to describe expression variability. Regardless of the radiation dose, we found the highest total and unique number of hypo- and hyper-variable genes in fibroblasts of cancer-free controls. Genes only classified as hypo-variable genes in this donor group were associated with regulatory processes and the stress response. Genes that were only hyper-variable in fibroblasts of childhood cancer survivors with at least one second primary neoplasm after the low dose were associated with the cell fate decision. In long-term survivors of childhood cancer without any second primary neoplasms, no prominent pathways were predicted to be uniquely affected by genes with hypo- or hyper-variable expression.

### Computation of bimodally expressed genes and EV

Bimodally expressed genes were either differentially expressed genes between donor groups or genes that showed a bimodal distribution across all donor groups. Interestingly, after LDIR, the total number of bimodal genes was far higher and the proportion of differentially expressed genes was lower than after the sham irradiation and HDIR. The observed substantial number of bimodal genes after LDIR may be explained by an intrinsic threshold for radiation-response, potentially irrespective of the donor group. Regarding genes that were not excluded due to their bimodal expression, the distribution of EV was similar across donor groups, but across all radiation doses, more genes were classified as hypo- and hyper-variable in N0 than in N1 and N2 + . N0 showed distinct and unique hypo-variable genes, which were associated with regulatory and stabilizing functions after all doses.

### Reaction to LDIR

Most notably, we identified genes to be only hyper-variable in N2 + post-LDIR that were associated with the cell fate decision. Among these were the *ERK1 and ERK2 cascade*, that are essential regulators of cell proliferation, differentiation, as well as the response to stress (Guo et al. 2020). Additionally, *RAS *protein signal transduction, also important for cell growth, division, and differentiation (Molina and Adjei 2006) and *Protein Kinase B signaling*, whereas its three isoforms are associated with the promotion of proliferation and increased cell survival (Nicholson and Anderson 2002), were identified exclusively for N2+ post-LDIR. We have previously reported that disturbances in the proper damage recognition and subsequent cell fate decision post-LDIR may be a cancer-driving factor in N2 + (Grandt et al. 2022). The best performing machine learning algorithm for the classification of N1 and N2 + based on the 30 genes that were only hyper-variable in N2 + post-LDIR showed an accuracy of only 0.68. We explain this due to the computational nature of the classification, since these usually rely on metrics such as mean, variance, and their relationship. As such metrics are, as shown by this work, sensitive to low expression levels and/or not the perfect fit for RNASeq-data distribution, novel algorithms might be needed to incorporate information such as the EV. However, since the EV is a metric that is computed on the group level, this is not straightforward to implement and requires further efforts. Interestingly, isoform one (*AKT1*) of *protein kinase B* was observed to halt apoptosis and induce cell-cycle progression only in N2 + through its upregulation post-HDIR (Grandt et al. 2022), further highlighting the role of the *protein kinase B* for radiation induced transfer of DNA-damage to the next cellular generations through incorrect cell fate decisions post-LDIR and -HDIR.

### Reaction to HDIR

The importance of the adequate regulation of the cell fate decision was further underlined by two findings. First, only in N0 positive regulation of cell cycle was a function of all hypo-variable and can be presumed to be only tightly regulated in N0. Second, in N1 and N2 + but not N0, regulation of fibroblast proliferation and fibroblast proliferation were GO terms over-represented in hyper-variable genes, and thus only deregulated in the long-term survivors of childhood cancer post-HDIR.

### Radiation-induced methylation

Genes that were reported to be affected by radiation-induced changes in methylation (Miousse et al. 2017) were either classified as non-variable or at least did not change classification across radiation treatments. Assuming that methylation has a radiation-dependent influence on EV and may be measured by our analysis pipeline presented, it is possible that 4 h after irradiation changes in global methylation may not be present in the early phase of DNA damage response (Maierhofer et al. 2017).

### Inter-individual variation and the response to ionizing radiation

Tumour treatment, and radiotherapy in particular, can lead to acute normal tissue toxicities or late effects such as secondary primary malignancies in long-term survivors of childhood cancer. The occurrence of acute radiogenic normal tissue toxicities is related to alterations of pathways such as DNA damage response, cell cycle, chromatin organization, and RNA metabolism, which are also thought to be responsible for radiogenic late effects (Gomolka et al. 2019). Thus, it is hypothesized that cellular processes span beyond these well-established pathways that regulate cellular survival after radiation (Yard et al. 2016). Although this study could not use data on immediate adverse reactions to ionizing radiation in donors such as inflammation of tissues, we observed donor group-specific variability in functions associated with adverse reactions to ionizing radiation to be hypo-variable only in fibroblasts of cancer-free controls.

### Strengths and limitations

While other studies examined small samples sizes (Storey et al. 2007) or used cell lines (Stranger et al. 2012) which are not able to fully depict the true variability in a population, we analysed a large donor group (N = 156) with a unique profile of cancer-free controls and childhood cancer survivors with and without second primary neoplasms. To reduce technical variation (commonly referred to as noise), all experiments were conducted in the same lab using matched triplets, consisting of one N0, one N1, and one N2 + donor. These triplets were then simultaneously subjected to the same treatment under identical lab conditions. Moreover, we used a pipeline that accounted for sampling error by incorporating bootstrapping and cross-validation in the classification process. The method used is more robust to outliers compared to other proposed methods (Storey et al. 2007; Li et al. 2010; Stranger et al. 2012). Despite all our efforts to reduce the technical variation to a minimum, we cannot fully exclude the possibility that other factors e.g., differences in sampling location (N2 + and N1 were predominantly sampled from inside the elbow by local dermatologists for this study, while N0 donors were predominantly sampled from the knee and feet in the course of elective surgery) between cancer survivors and tumour-free donors may have had an impact on expressional variability. Nevertheless, we expect that filtering of bimodal genes excluded such genes (e.g., the various *HOX*-cluster and their expression dependent on the developmental axes (Rinn et al. 2007)). We compared identical cell types and matched the donors by central parameters that affect EV, such as sex and age, which have been previously shown to be the most influential factor for the EV (Bashkeel et al. 2019), to successfully identify subtle group differences in EV between donor groups that may be associated with their respective cancer status, especially via sex-specific modulation of immune-pathways as one recent work reported (Khodursky et al. 2022). Thus, we applied analyses stratified by sex to examine these potential differences in more detail. Our analyses did identify sex-specific differences in the number of bimodally expressed genes, but not in EV and its subsequent variability classification. A difference in bimodal expression is unsurprising as the sex-specific expression can be found across all tissues and are of immense pathophysiological relevance (Gershoni and Pietrokovski 2017) and impact the radiation response (Narendran et al. 2019). To further elaborate on this, we further accounted for EV-modulating factors such as smoking and drinking habits in the additional sensitivity analyses. Smoking and excessive consumption of alcohol have been shown to affect gene expression in active vs never smokers (Cao et al. 2015), as well as smokers compared to non-tobacco consumers (Arimilli et al. 2017). However, the genes proposed as biomarkers for smoking individuals (*CYP1A1*, *CYP1B1*, *YWHAZ* and *PTPRD*, *MAX*, and *USF1*) did not differ in classification between the samples termed healthy and those termed unhealthy, irrespective of the radiation dose. Nevertheless, further work with more adequate sample sizes is needed to elaborate on these findings.

## Conclusion

For the first time, we adapted a comprehensive method to compute and characterize the expression variability unaffected by the expression magnitude in fibroblasts of childhood cancer survivors and cancer-free controls in response to a low and a high dose of ionizing radiation. Our results suggest that cancer-free controls exhibit different variability structure in gene expression with more hypo- and hyper-variable genes than long-term survivors of childhood cancer. The fibroblasts of these former childhood cancer patients with at least one second primary neoplasm showed deregulated mechanisms essential for cell fate decision. This could partially explain a subsequently occurring second primary neoplasm. Based on these results we encourage future works to focus on pathways for cell fate decision post-LDIR to shed further light on the carcinogenesis of potentially radiation induced second primary neoplasms.

## Supplementary Information

**Additional file 1.** Results of the differential gene expression analysis and the bimodal indices. a: Results of the differential expression analysis comparing fibroblasts of long-term survivors of childhood cancer without (N1) and with at least one second primary neoplasm (N2 +) to cancer-free controls (N0). Data were adjusted for age and sex, p-values for false discovery at a rate of 0.05 (FDR). b: Bimodal index (BI) values of the bimodal test, using the SIBERG package for R.

**Additional file 2.** Results of the gene expression variability classification. a Gene classifications in whole data set (WDS) and classifications after cross-validation (cv) in fibroblasts of cancer-free controls (N0), childhood cancer survivors without (N1) and with at least one second primary neoplasm (N2 +) after exposure 0 Gray. 1 = Hypo-Variable, 2 = Non-Variable, 3 = Hyper-Variable. NA = Gene excluded by bimodal test/NA in CV = classification from WDS not verified, set to 2 for further analyses. b Gene classifications in whole data set (WDS) and classifications after cross-validation (cv) in fibroblasts of cancer-free controls (N0), long-term survivors of childhood cancer without (N1) and with at least one second primary neoplasm (N2 +) after exposure to 0.05 Gray. 1 = Hypo-Variable, 2 = Non-Variable, 3 = Hyper-Variable. NA = Gene excluded by bimodal test/NA in CV = classification from WDS not verified, set to 2 for further analyses. c Gene classifications in whole data set (WDS) and classifications after cross-validation (cv) in fibroblasts of cancer-free controls (N0), long-term survivors of childhood cancer without (N1) and with at least one second primary neoplasm (N2 +) after exposure to 2 Gray. 1 = Hypo-Variable, 2 = Non-Variable, 3 = Hyper-Variable. NA = Gene excluded by bimodal test/NA in CV = classification from WDS not verified, set to 2 for further analyses. d Results of the various employed algorithms for the classification of N1 and N2 + using 30 genes that were only classified as hyper-variable in N2 + post-LDIR. Models were trained and tested using only N1 and N2 + data post-LDIR.

**Additional file 3.** Gene lists that were used as backgrounds in the gene ontology over-representation analyses.

**Additional file 4.** Results of the Gene Ontology (GO) term over-representation analyses. Results of the Gene Ontology (GO) term over-representation analyses for all donor groups and radiation doses, filtered for adjusted *p*-value < 0.05. N0 = fibroblasts of cancer-free donors, N1 = fibroblasts of long-term survivors of childhood cancer without a second primary neoplasm, and N2 +  = fibroblasts of long-term survivors of childhood cancer with at least one second primary neoplasm.

**Additional file 5.** Intersect graphs of A) genes and B) Gene Ontology (GO) terms. Intersect graphs of A) genes and B) Gene Ontology (GO) terms: Both graphs are stratified by variability-classification, donor group (N0 = fibroblasts of cancer-free controls, N1 = fibroblasts of childhood cancer survivors without a second primary neoplasm, N2 +  = fibroblasts of childhood cancer survivors with at least one second primary neoplasm), and radiation dose. Connected rows implicate that A) genes or B) GO terms were identically classified in these data. The bars denote the summed number of identically classified A) genes or B) GO terms among the vertically connected rows of data, e.g., in A) the first column implicates that 416 genes were found to be hypo-variable across all radiation doses and donor groups.

**Additional file 6.** Gene lists that were used as backgrounds in the gene ontology over-representation analyses. a: Over-represented Gene Ontology terms for genes only classified hypo-variable (n = 49) in fibroblasts of cancer-free donors after 0 Gray. b: Over-represented Gene Ontology terms for genes classified only hypo-variable (n = 41) in fibroblasts of cancer-free controls after 0.05 Gray. c: Over-represented Gene Ontology terms for genes classified only hypo-variable (n = 38) in fibroblasts of cancer-free donors after 2 Gray. d: Over-represented Gene Ontology terms for genes classified only as hyper-variable (n = 43) in fibroblasts of cancer-free donors after all radiation doses. e: Over-represented Gene Ontology terms for genes classified only hypo-variable (n = 30) in fibroblasts of long-term survivors of childhood cancer with at least one second primary neoplasm after 2 Gray. f: Over-represented Gene Ontology terms for genes classified only hypo-variable (n = 30) in fibroblasts of long-term survivors of childhood-cancer with at least one second primary neoplasm after 0.05 Gray, filtered for the 14 genes with informational value for classification discrimination between N1 and N2 + .

**Additional file 7.** Comparison of the bimodally expressed genes with the information on differential gene expression. Comparison of differential gene expression analysis and results of the bimodal test. A.) Volcano plots comparing bimodal index values and log_2_ fold-change values, stratified by radiation dose. Here, the expression of fibroblasts of cancer groups (N1 = fibroblasts of long-term survivors of childhood cancer without a second primary neoplasm, N2 +  = fibroblasts of long-term survivors of childhood cancer with at least one second primary neoplasm.) was compared to those of cancer-free controls. The dashed line indicates the threshold for bimodal expression set to bimodal index = 1.1. B.) Bar charts showing the total number of bimodally expressed genes per radiation dose and the number of differentially expressed genes thereof per comparison.

**Additional file 8.** Stratified_Analyses. a: Violin and jitter plots comparing results of the analyses for bimodally expressed genes stratified by radiation dose and sex. With respect to the reduced sample size due to the stratification, the cut-off was increased to 1.3 (dashed line). Nevertheless, these sample sizes (female: n = 81, male: n = 75) could still not have been sufficient to provide adequate power for the detection of bimodally expressed genes. b: Comparison of expressional variation by sex. The scatterplot compares the sex-specific EV per gene and radiation dose. The black line indicates the linear regression model (adjusted r^2^: 0.779; Kendall’s tau: 0.786). Only genes with a bimodal index < 1.3 were included in the analysis. c: Bar charts showing the number of genes per classification using the whole data set and the number of genes with stable classification after cross-validation per radiation dose. Data shown here were stratified by sex. Only genes with a bimodal index < 1.3 were included in the analyses. N0 = fibroblasts of cancer-free controls, N1 = fibroblasts of long-term survivors of childhood cancer without a second primary neoplasm, and N2 +  = fibroblasts of long-term survivors of childhood cancer with at least one second primary neoplasm. d: Intersect graphs of overlapping gene classifications: Data are stratified by variability classification, donor group (N0 = fibroblasts of cancer-free controls, N1 = fibroblasts of long-term survivors of childhood cancer without a second primary neoplasm, N2 +  = fibroblasts of long-term survivors of childhood cancer with at least one second primary neoplasm), sex, and radiation dose. Connected rows implicate that genes were identically classified in these data sets. Bars denote the summed number of identically classified genes among the vertically connected rows of data. e: Violin and jitter plots comparing results of the analyses for bimodally expressed genes stratified by radiation dose and consumption information on smoking and alcohol. Data used here were donor triplets without heavy smokers (> 10 pack years) and/or alcohol consumption (> 2 alcoholic beverages per day), termed “healthy” for brevity; as well as triplets that contained at least one donor with the above-described lifestyle, shortly termed “unhealthy”. To ensure validity, the cut-off was increased to 1.3 due to the reduced sample size. Nevertheless, these sample sizes (Donors from triplets without any heavy smokers or drinkers: n = 60, donors from triplets with at least one heavy drinker or smoker: n = 54) might not be sufficient to provide adequate power for the detection of bimodally expressed genes with the given cut-off. f: Bar charts showing the number of genes per classification using the whole data set and the number of genes with stable classification after cross-validation per radiation dose. Data used here were donor triplets without heavy smokers (> 10 pack years) and/or alcohol consumption (> 2 alcoholic beverages per day), termed”healthy”; as well as triplets that contained at least one donor with the above-described lifestyle, termed “unhealthy” for brevity. Only genes with a bimodal index < 1.3 were included in the analyses. N0 = fibroblasts of cancer-free controls, N1 = fibroblasts of long-term survivors of childhood cancer without a second primary neoplasm, and N2 +  = fibroblasts of long-term survivors of childhood cancer with at least one second primary neoplasm. g: Comparison of the EV per gene and radiation dose comparison donor triplets with and without heavy smokers (> 10 pack years) and/or alcohol consumption (> 2 alcoholic beverages per day). The red line indicates the linear regression model (adjusted r^2^: 0.377; Kendall’s tau: 0.683). Only genes with a bimodal index < 1.3 were included in the analysis. h: Intersect graphs of overlapping gene classifications: Data are stratified by variability classification, donor group (N0 = fibroblasts of cancer-free controls, N1 = fibroblasts of long-term survivors of childhood cancer without a second primary neoplasm, N2 +  = fibroblasts of long-term survivors of childhood cancer with at least one second primary neoplasm), radiation dose, and additionally computed using only the 20 donor triplets (n = 60) without heavy smokers (> 10 pack years) and/or alcohol consumption (> 2 alcoholic beverages per day). Connected rows implicate that genes were identically classified in these data sets. Bars denote the summed number of identically classified genes among the vertically connected rows of data.

**Additional file 9.** Comparison of the bimodally expressed genes with the information on differential gene expression. Expression variation and variability classification of genes that were presumed to be affected in methylation status by ionizing radiation in the literature, stratified by donor group and radiation dose. N0 = fibroblasts of cancer-free donors, N1 = fibroblasts of long-term survivors of childhood cancer without a second primary neoplasm, and N2 +  = fibroblasts of long-term survivors of childhood cancer with at least one second primary neoplasm.

## Data Availability

The datasets used and/or analysed during the current study are available from the corresponding author upon reasonable request. An exemplary code to reproduce the classification pipeline can be found on the GitHub-repository: https://github.com/clg1990/KiKme_mrna_variability.

## Abbreviations

ABCF3: Atp Binding Cassette Subfamily F Member 3
ALDOA: Aldolase, Fructose-Bisphosphate A
ANGPT1: Angiopoietin 1
ANPEP: Alanyl Aminopeptidase, Membrane
ARID1A: At-Rich Interaction Domain 1a
ASPH: Aspartate Beta-Hydroxylase
ATF4: Activating Transcription Factor 4
BAG5: Bag Cochaperone 5
CAMK1D: Calcium/Calmodulin Dependent Protein Kinase Id
CCM2: Ccm2 Scaffold Protein
CCND1: Cyclin D1
CCNG1: Cyclin G1
CD44: Cd44 Molecule (Indian Blood Group)
CD63: Cd63 Molecule
CDH13: Cadherin 13
CDKN1A: Cyclin Dependent Kinase Inhibitor 1a
CIRBP: Cold Inducible Rna Binding Protein
CST3: Cystatin C
CV: Coefficient of variation
CYP1A1: Cytochrome P450 Family 1 Subfamily A Member 1
CYP1B1: Cytochrome P450 Family 1 Subfamily B Member 1
DKK2: Dickkopf Wnt Signaling Pathway Inhibitor 2
DNA: Deoxy-Ribonucleic Acid
DNAJB2: Dnaj Heat Shock Protein Family (Hsp40) Member B2
EIF2AK2: Eukaryotic Translation Initiation Factor 2 Alpha Kinase 2
ENO1: Enolase 1
EV: Expression variability
ERK1: Ras-dependent extracellular signal-regulated kinase 1
ERK2: Ras-dependent extracellular signal-regulated kinase 2
FBLN5: Fibulin 5
FGF5: Fibroblast growth factor 5
FH: Fumarate Hydratase
GLB1: Galactosidase Beta 1
GLUL: Glutamate-Ammonia Ligase
GNAQ: G Protein Subunit Alpha Q
GO: Gene Ontology
GTF2H1: General Transcription Factor IIH Subunit 1
HDIR: High Dose Ionizing Radiation
IGFBP3: Insulin Like Growth Factor Binding Protein 3
IL1R1: Interleukin 1 Receptor Type 1
IL6ST: Interleukin 6 Cytokine Family Signal Transducer
IMPDH2: Inosine Monophosphate Dehydrogenase 2
KLF2: Krüppel Like Factor 2
LAMB2: Laminin Subunit Beta 2
LASP1: Lim And Sh3 Protein 1
LDIR: Low Dose Ionizing Radiation
LIMS1: Lim Zinc Finger Domain Containing 1
LOXL3: Lysyl Oxidase Like 3
LRP1: LDL Receptor Related Protein 1
MAD: Median Absolute Deviation
MAPK: Mitogen-activated protein kinase
MAX: Myc Associated Factor X
mRNA: Messenger Ribonucleic Acid
MSH6: Muts Homolog 6
MSRB3: Methionine Sulfoxide Reductase B3
MTCH1: Mitochondrial Carrier 1
MXRA8: Matrix Remodeling Associated 8
MYH10: Myosin Heavy Chain 10
N0: Fibroblasts of cancer-free Controls
NRP1: Neuropilin-1
N1: Fibroblasts of Donors with a First Primary Neoplasm in Childhood
N2 +: Fibroblasts of Donors with a First Primary Neoplasm in Childhood and at least one Second Primary Neoplasm
OSMR: Oncostatin M Receptor
PLS3: Plastin 3
POLR2C: Rna Polymerase II Subunit C
PPIG: Peptidylprolyl Isomerase G
PPP1R10: Protein Phosphatase 1 Regulatory Subunit 10
PRDX5: Peroxiredoxin 5
PSMA3: Proteasome 20 s Subunit Alpha 3
PSMB3: Proteasome 20 s Subunit Beta 3
PSMD4: Proteasome 26 s Subunit Ubiquitin Receptor, Non-Atpase 4
PSMD5: Proteasome 26 s Subunit, Non-ATPase 5
PTPRD: Protein Tyrosine Phosphatase Receptor Type D
RAD21: Rad21 Cohesin Complex Component
RAD23A: Rad23 Homolog A, Nucleotide Excision Repair Protein
RAD23B: Rad23 Homolog B, Nucleotide Excision Repair Protein
RALGPS2: Ral GEF with PH Domain and SH3 Binding Motif 2
Ras: Rat sarcoma virus
RCN3: Reticulocalbin 3
ROBO1: Roundabout Guidance Receptor 1
RPLP0: Ribosomal Protein Lateral Stalk Subunit P0
RPS18: Ribosomal Protein S18
RPS19: Ribosomal Protein S19
RPS19: Ribosomal Protein S19
RPS27L: Ribosomal Protein S27 Like
RRAS: Ras-related Protein R-Ras
SD: Standard deviation
SIGMAR1: Sigma Non-Opioid Intracellular Receptor 1
SIGMAR1: Sigma Non-Opioid Intracellular Receptor 1
SIN3A: Sin3 Transcription Regulator Family Member A
SIN3A: Sin3 Transcription Regulator Family Member A
SPEN: Spen Family Transcriptional Repressor
SPTBN1: Spectrin Beta, Non-Erythrocytic 1
SREBF2: Sterol Regulatory Element Binding Transcription Factor 2
SRPK2: SRSF Protein Kinase 2
STK25: Serine/Threonine Kinase 25
STK25: Serine/Threonine Kinase 25
THY1: Thy-1 Cell Surface Antigen
TMEM119: Transmembrane Protein 119
TNFAIP8L3: TNF Alpha Induced Protein 8 Like 3
TOLLIP: Toll Interacting Protein
TRAF7: TNF Receptor Associated Factor 7
TRAIL: TNF-related apoptosis-inducing Ligand
TRAM2: Translocation Associated Membrane Protein 2
TXNRD1: Thioredoxin Reductase 1
TXNRD1: Thioredoxin Reductase 1
UBB: Ubiquitin B
ULK1: Unc-51 Like Autophagy Activating Kinase 1
USF1: Upstream Transcription Factor 1
VDAC3: Voltage Dependent Anion Channel 3
WDR48: WD Repeat Domain 48
YBX3: Y-Box Binding Protein 3
YWHAB: Tyrosine 3-Monooxygenase/Tryptophan 5-Monooxygenase Activation Protein Beta
YWHAE: Tyrosine 3-Monooxygenase/Tryptophan 5-Monooxygenase Activation Protein Epsilon
YWHAG: Tyrosine 3-Monooxygenase/Tryptophan 5-Monooxygenase Activation Protein Gamma
YWHAQ: Tyrosine 3-Monooxygenase/Tryptophan 5-Monooxygenase Activation Protein Theta
YWHAZ: Tyrosine 3-Monooxygenase/Tryptophan 5-Monooxygenase Activation Protein Zeta
ZMIZ1: Zinc Finger Miz-Type Containing 1
