# Supplementary figures and images for: Gene expression variability in long-term survivors of childhood cancer and cancer-free controls in response to ionizing irradiation
Source: Mol Med. 2023 Mar 30;29:41. doi: 10.1186/s10020-023-00629-2 (PMC10061869; doi:10.1186/s10020-023-00629-2)

# A

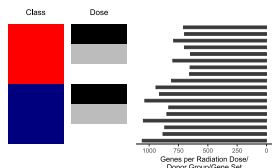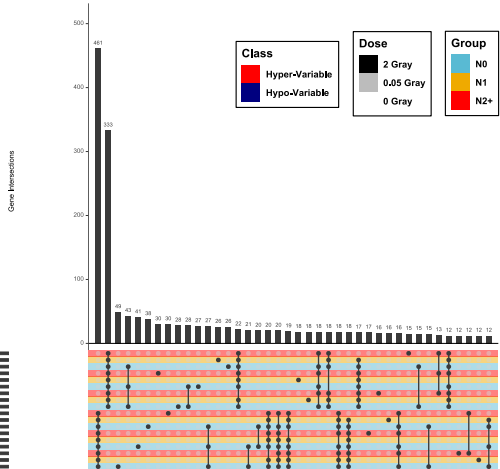

# B

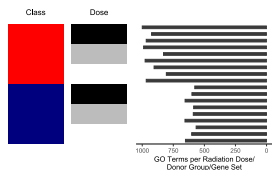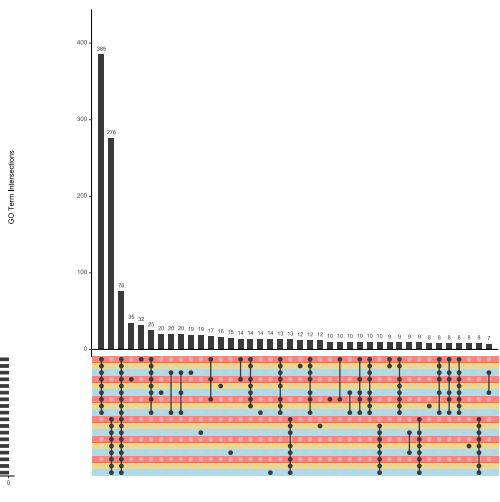

Supplement: Supplementary file 5 — Additional file 5. Intersect graphs of A) genes and B) Gene Ontology (GO) terms. Intersect graphs of A) genes and B) Gene Ontology (GO) terms: Both graphs are stratified by variability-classification, donor group (N0 = fibroblasts of cancer-free controls, N1 = fibroblasts of childhood cancer survivors without a second primary neoplasm, N2 + = fibroblasts of childhood cancer survivors with at least one second primary neoplasm), and radiation dose. Connected rows implicate that A) genes or B) GO terms were identically classified in these data. The bars denote the summed number of identically classified A) genes or B) GO terms among the vertically connected rows of data, e.g., in A) the first column implicates that 416 genes were found to be hypo-variable across all radiation doses and donor groups. [file 10020_2023_629_MOESM5_ESM.pdf]

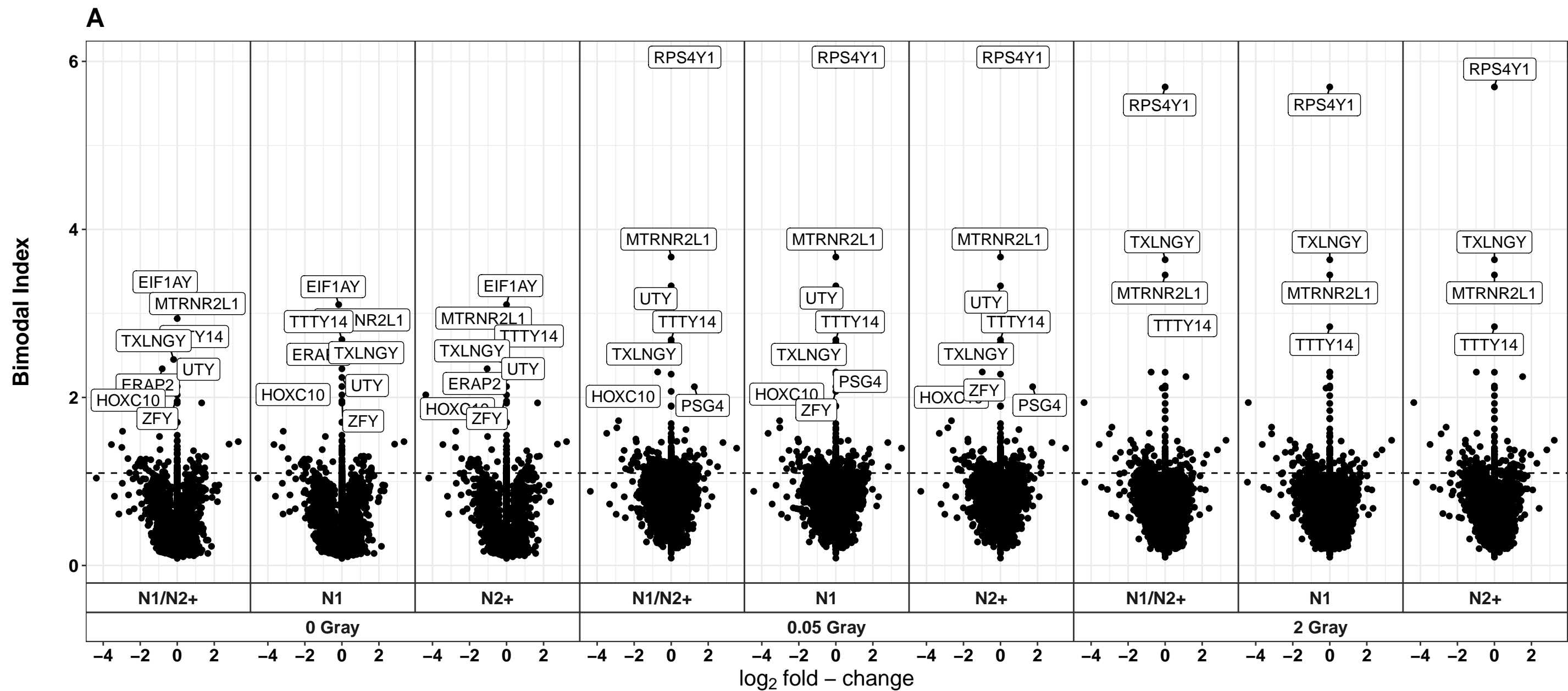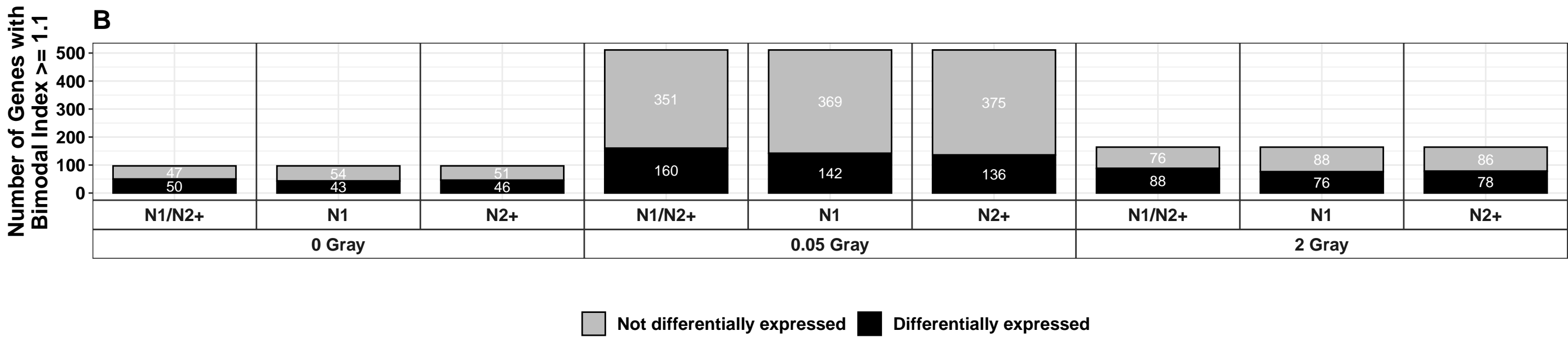

Supplement: Supplementary file 7 — Additional file 7. Comparison of the bimodally expressed genes with the information on differential gene expression. Comparison of differential gene expression analysis and results of the bimodal test. A.) Volcano plots comparing bimodal index values and log2 fold-change values, stratified by radiation dose. Here, the expression of fibroblasts of cancer groups (N1 = fibroblasts of long-term survivors of childhood cancer without a second primary neoplasm, N2 + = fibroblasts of long-term survivors of childhood cancer with at least one second primary neoplasm.) was compared to those of cancer-free controls. The dashed line indicates the threshold for bimodal expression set to bimodal index = 1.1. B.) Bar charts showing the total number of bimodally expressed genes per radiation dose and the number of differentially expressed genes thereof per comparison. [file 10020_2023_629_MOESM7_ESM.pdf]

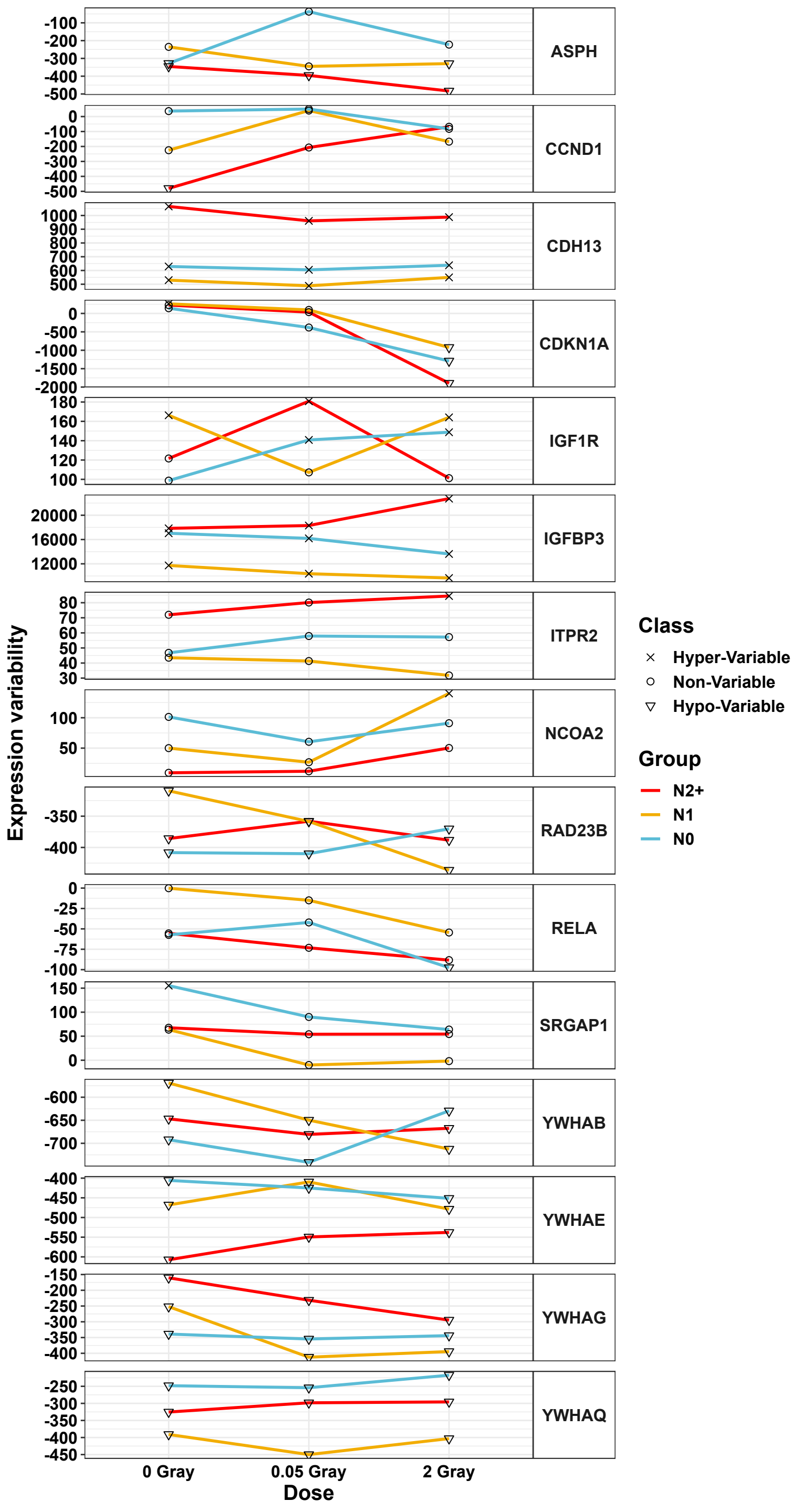

Supplement: Supplementary file 9 — Additional file 9. Comparison of the bimodally expressed genes with the information on differential gene expression. Expression variation and variability classification of genes that were presumed to be affected in methylation status by ionizing radiation in the literature, stratified by donor group and radiation dose. N0 = fibroblasts of cancer-free donors, N1 = fibroblasts of long-term survivors of childhood cancer without a second primary neoplasm, and N2 + = fibroblasts of long-term survivors of childhood cancer with at least one second primary neoplasm. [file 10020_2023_629_MOESM9_ESM.pdf]
