# Supplementary material for: Gene expression variability in long-term survivors of childhood cancer and cancer-free controls in response to ionizing irradiation
Source: Mol Med. 2023 Mar 30;29:41. doi: 10.1186/s10020-023-00629-2 (PMC10061869; doi:10.1186/s10020-023-00629-2)
Supplement: Supplementary file 6 — Additional file 6. Gene lists that were used as backgrounds in the gene ontology over-representation analyses. a: Over-represented Gene Ontology terms for genes only classified hypo-variable (n = 49) in fibroblasts of cancer-free donors after 0 Gray. b: Over-represented Gene Ontology terms for genes classified only hypo-variable (n = 41) in fibroblasts of cancer-free controls after 0.05 Gray. c: Over-represented Gene Ontology terms for genes classified only hypo-variable (n = 38) in fibroblasts of cancer-free donors after 2 Gray. d: Over-represented Gene Ontology terms for genes classified only as hyper-variable (n = 43) in fibroblasts of cancer-free donors after all radiation doses. e: Over-represented Gene Ontology terms for genes classified only hypo-variable (n = 30) in fibroblasts of long-term survivors of childhood cancer with at least one second primary neoplasm after 2 Gray. f: Over-represented Gene Ontology terms for genes classified only hypo-variable (n = 30) in fibroblasts of long-term survivors of childhood-cancer with at least one second primary neoplasm after 0.05 Gray, filtered for the 14 genes with informational value for classification discrimination between N1 and N2 + . [file 10020_2023_629_MOESM6_ESM.docx]

**
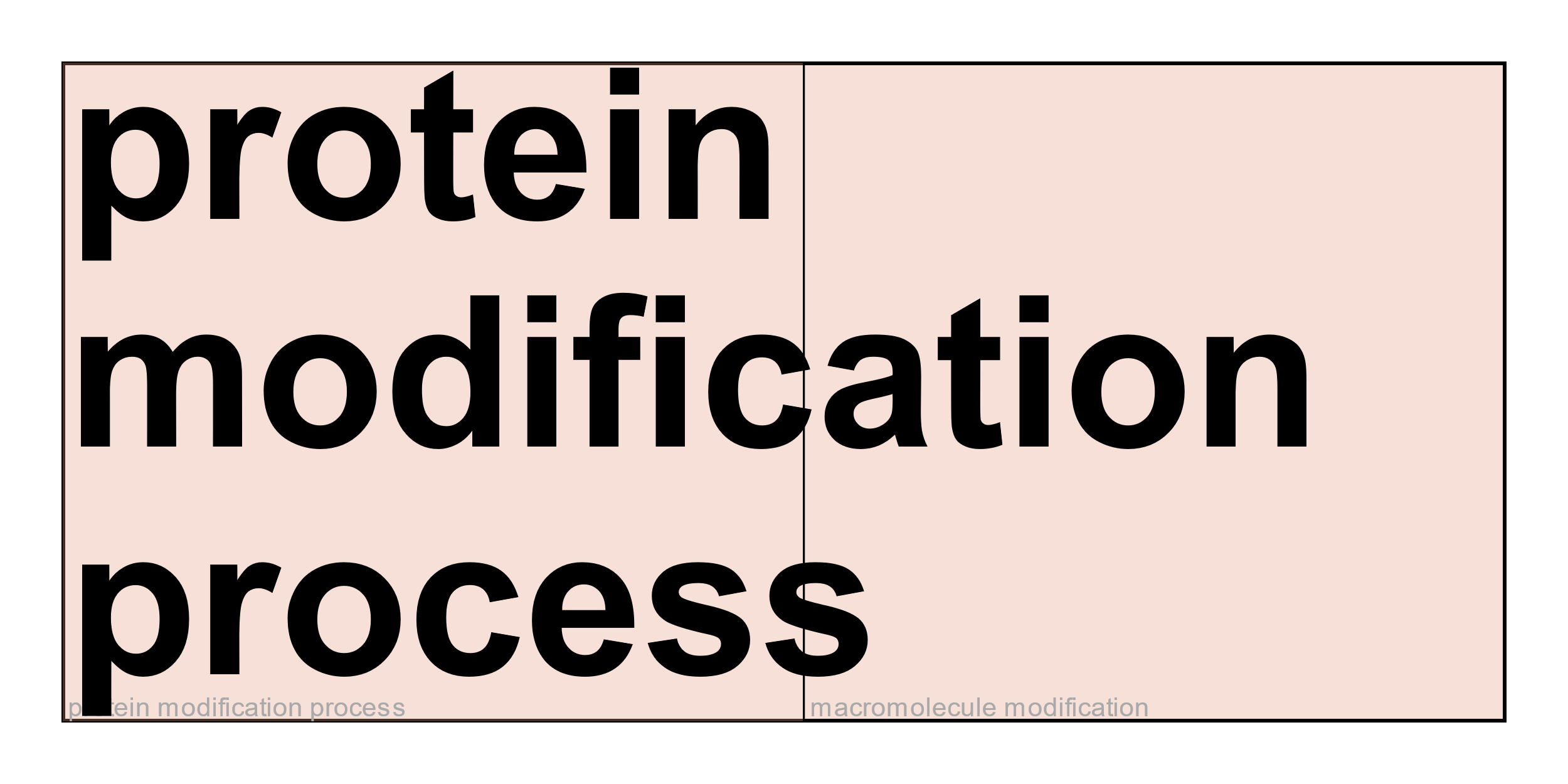
**

**Additional File 6a:** Over-represented Gene Ontology terms for genes only classified hypo-variable (n=49) in fibroblasts of cancer-free donors after 0 Gray.

**
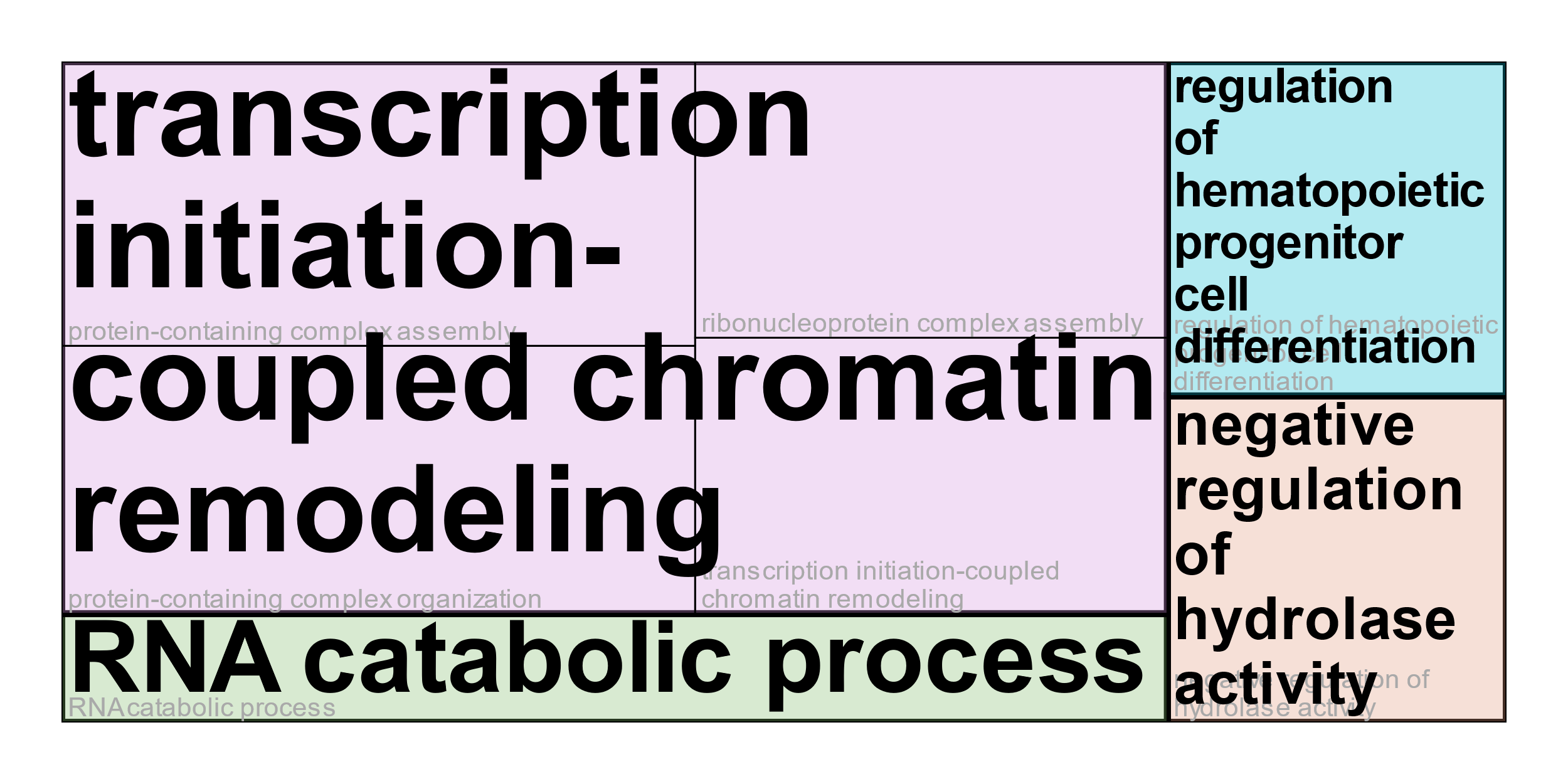
**

**Additional File 6b:** Over-represented Gene Ontology terms for genes classified only hypo-variable (n=41) in fibroblasts of cancer-free donors after 0.05 Gray.

**
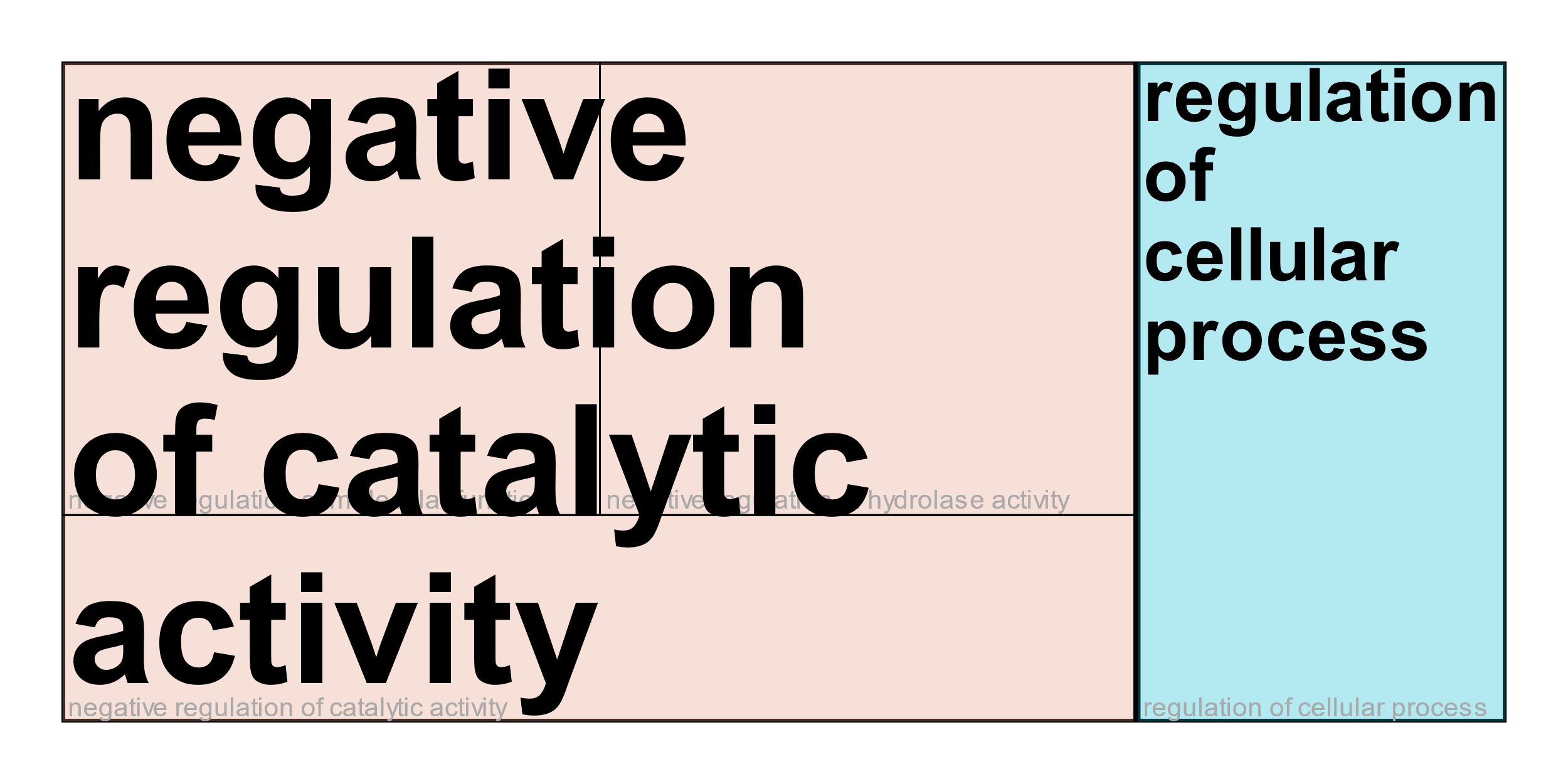
**

**Additional File 6c:** Over-represented Gene Ontology terms for genes classified only hypo-variable (n=38) in fibroblasts of cancer-free donors after 2 Gray.

**
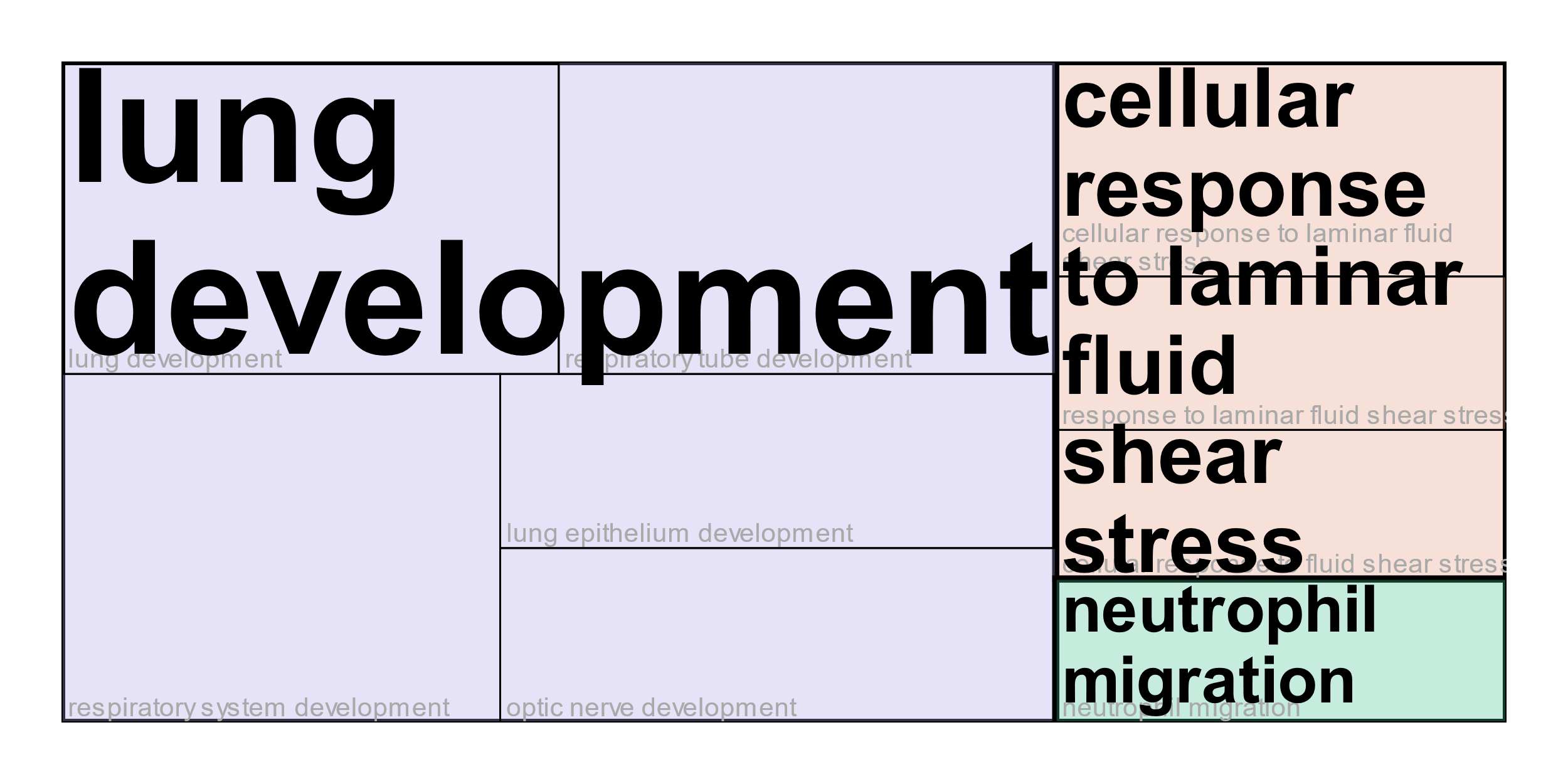
Additional File 6d:** Over-represented Gene Ontology terms for genes classified only as hyper-variable (n=43) in fibroblasts of cancer-free donors after all radiation doses.

**
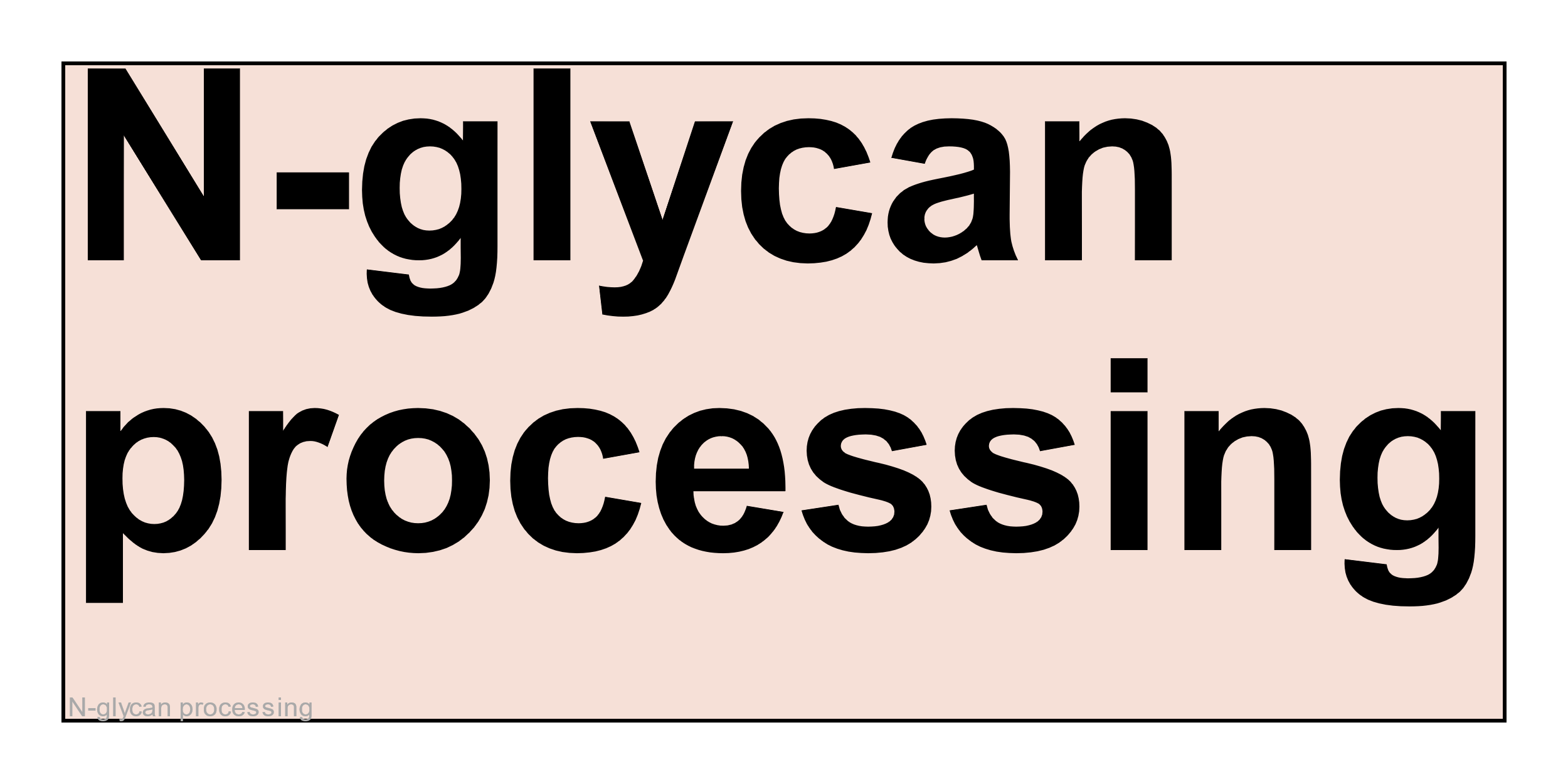
**

**Additional File 6e:** Over-represented Gene Ontology terms for genes classified only hypo-variable (n=30) in fibroblasts of long-term survivors of childhood-cancer with at least one second primary neoplasm after 2 Gray.


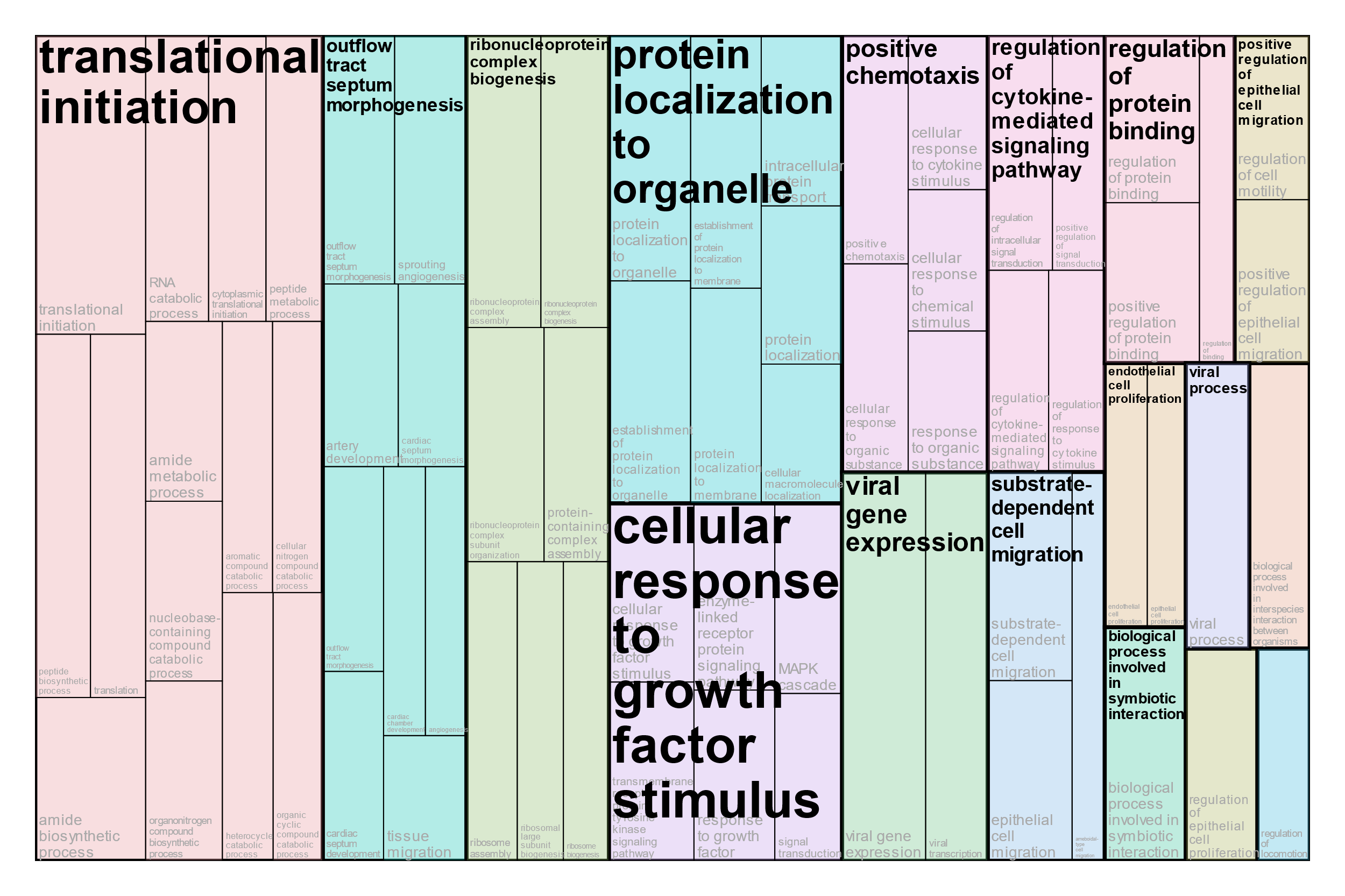


**Additional File 6f:** Over-represented Gene Ontology terms for genes classified only hypo-variable (n=30) in fibroblasts of long-term survivors of childhood-cancer with at least one second primary neoplasm after 0.05 Gray, filtered for the 14 genes with informational value for classification discrimination between N1 and N2+.
