# Supplementary material for: Gene expression variability in long-term survivors of childhood cancer and cancer-free controls in response to ionizing irradiation
Source: Mol Med. 2023 Mar 30;29:41. doi: 10.1186/s10020-023-00629-2 (PMC10061869; doi:10.1186/s10020-023-00629-2)
Supplement: Supplementary file 8 — Additional file 8. Stratified_Analyses. a: Violin and jitter plots comparing results of the analyses for bimodally expressed genes stratified by radiation dose and sex. With respect to the reduced sample size due to the stratification, the cut-off was increased to 1.3 (dashed line). Nevertheless, these sample sizes (female: n = 81, male: n = 75) could still not have been sufficient to provide adequate power for the detection of bimodally expressed genes. b: Comparison of expressional variation by sex. The scatterplot compares the sex-specific EV per gene and radiation dose. The black line indicates the linear regression model (adjusted r2: 0.779; Kendall’s tau: 0.786). Only genes with a bimodal index < 1.3 were included in the analysis. c: Bar charts showing the number of genes per classification using the whole data set and the number of genes with stable classification after cross-validation per radiation dose. Data shown here were stratified by sex. Only genes with a bimodal index < 1.3 were included in the analyses. N0 = fibroblasts of cancer-free controls, N1 = fibroblasts of long-term survivors of childhood cancer without a second primary neoplasm, and N2 + = fibroblasts of long-term survivors of childhood cancer with at least one second primary neoplasm. d: Intersect graphs of overlapping gene classifications: Data are stratified by variability classification, donor group (N0 = fibroblasts of cancer-free controls, N1 = fibroblasts of long-term survivors of childhood cancer without a second primary neoplasm, N2 + = fibroblasts of long-term survivors of childhood cancer with at least one second primary neoplasm), sex, and radiation dose. Connected rows implicate that genes were identically classified in these data sets. Bars denote the summed number of identically classified genes among the vertically connected rows of data. e: Violin and jitter plots comparing results of the analyses for bimodally expressed genes stratified by radiation dose and consumption in [file 10020_2023_629_MOESM8_ESM.docx]

**
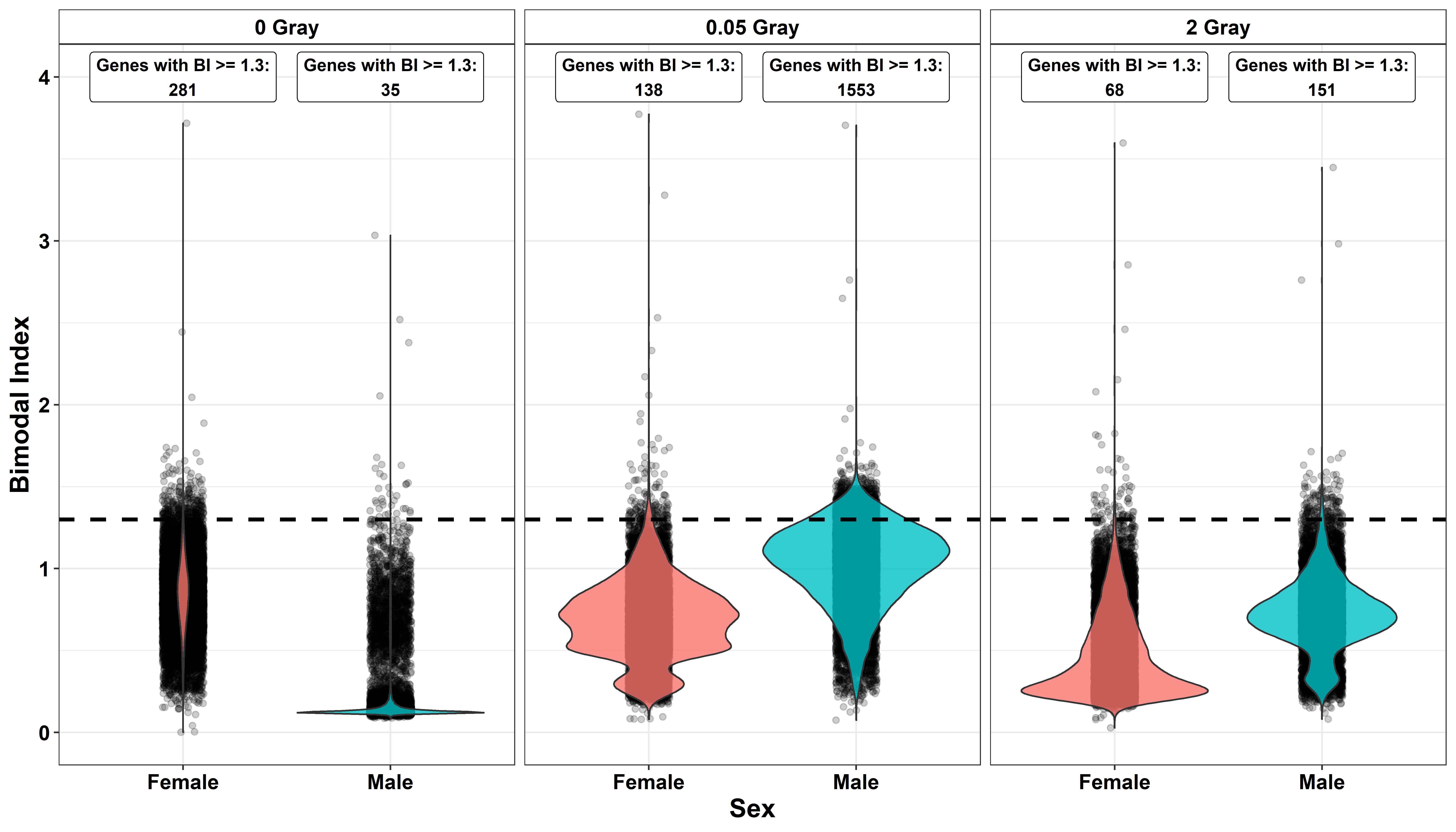
**

**Additional File 07a:** Violin and jitter plots comparing results of the analyses for bimodally expressed genes stratified by radiation dose and sex. With respect to the reduced sample size due to the stratification, the cut-off was increased to 1.3 (dashed line). Nevertheless, these sample sizes (female: n=81, male: n=75) could still not have been sufficient to provide adequate power for detection of bimodally expressed genes.


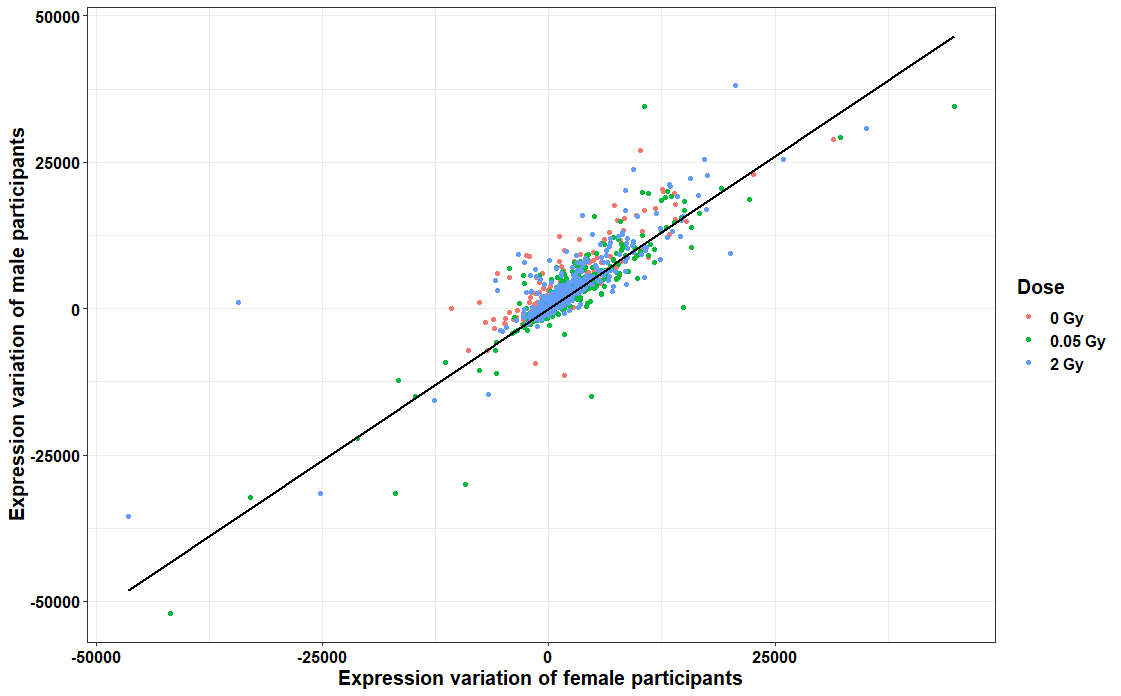


**Additional File 07b:** Comparison of expressional variation by sex. The scatterplot compares the sex-specific EV per gene and radiation dose. The black line indicates the linear regression model (**adjusted r²**: 0.779; **Kendall’s tau**: 0.786). Only genes with a bimodal index < 1.3 were included in the analysis.

**

**

**Additional File 7c**: Bar charts showing number of genes per classification using the whole data set and number of genes with stable classification after cross-validation per radiation dose. Data shown here were stratified by sex. Only genes with a bimodal index < 1.3 were included in the analyses. N0 = fibroblasts of cancer-free controls, N1 = fibroblasts of childhood cancer survivors without a second primary neoplasm, N2+ = fibroblasts of childhood cancer survivors with at least one second primary neoplasm.





**Additional Files 7d:** Intersect graphs of overlapping gene classifications: Data are stratified by variability-classification, donor group (N0 = fibroblasts of cancer-free controls, N1 = fibroblasts of childhood cancer survivors without a second primary neoplasm, N2+ = fibroblasts of childhood cancer survivors with at least one second primary neoplasm), sex, and radiation dose. Connected rows implicate that genes were identically classified in these data sets. Bars denote the summed number of identically classified genes among the vertically connected rows of data.


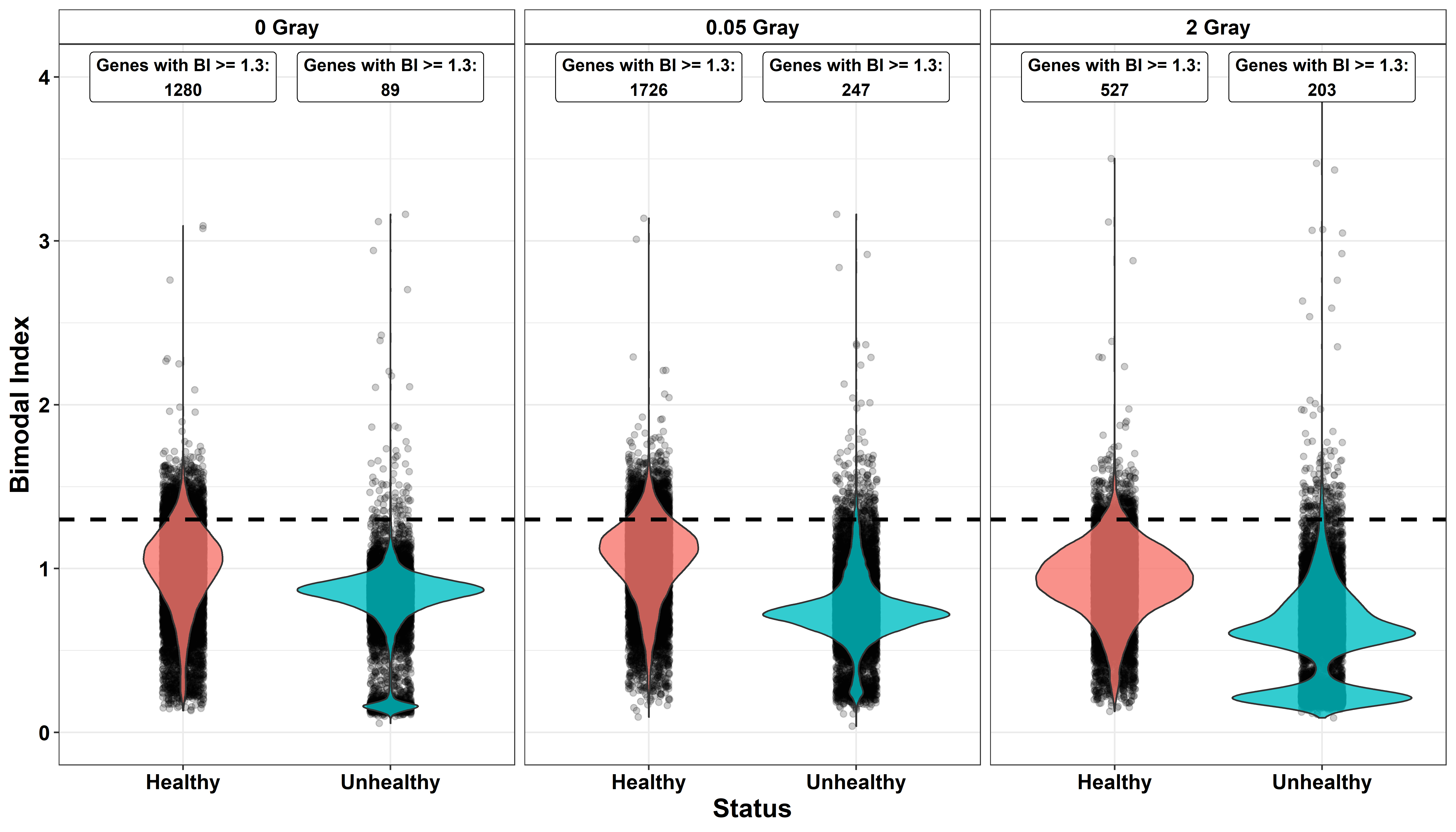


**Additional File 07e:** Violin and jitter plots comparing results of the analyses for bimodally expressed genes stratified by radiation dose and consumption information on smoking and alcohol. Data used here were donor triplets without heavy smokers (>10 pack years) and/or alcohol consumption (> 2 alcoholic beverages per day), termed ”healthy” for brevity; as well as triplets which contained at least one donor with the above-described lifestyle, shortly termed “unhealthy”. To ensure validity, the cut-off was increased to 1.3 due to the reduced sample size. Nevertheless, these sample sizes (Donors from triplets without any heavy smokers or drinkers: n=60, donors from triplets with at least one heavy drinker or smoker: n=54) might not be sufficient to provide adequate power for detection of bimodally expressed genes with the given cut-off.





**Additional File 7f**: Bar charts showing number of genes per classification using the whole data set and number of genes with stable classification after cross-validation per radiation dose. Data used here were donor triplets without heavy smokers (>10 pack years) and/or alcohol consumption (> 2 alcoholic beverages per day), termed ”healthy”; as well as triplets which contained at least one donor with the above-described lifestyle, termed “unhealthy” for brevity. Only genes with a bimodal index < 1.3 were included in the analyses. N0 = fibroblasts of cancer-free controls, N1 = fibroblasts of childhood cancer survivors without a second primary neoplasm, N2+ = fibroblasts of childhood cancer survivors with at least one second primary neoplasm.


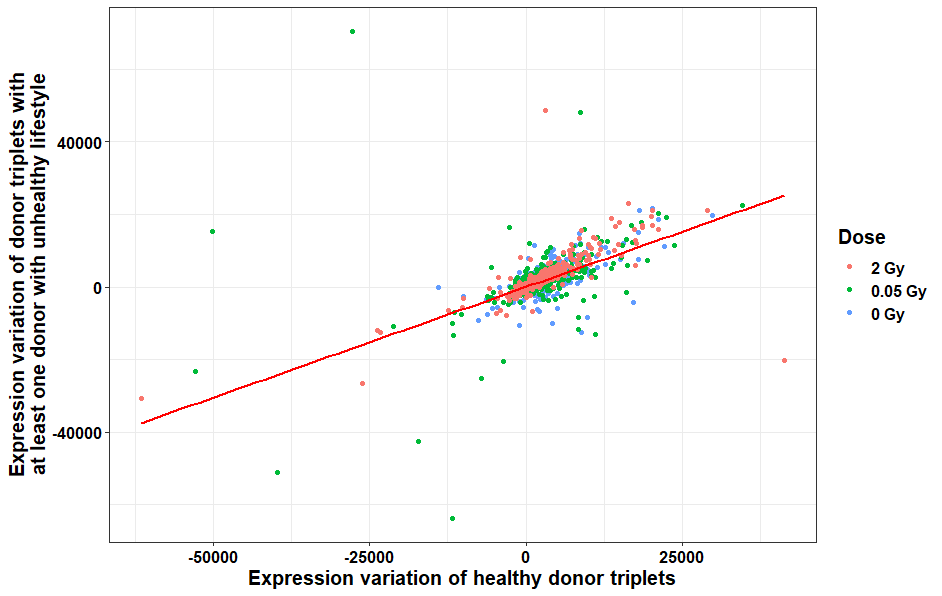
**Additional File 7g:** Comparison of the EV per gene and radiation dose comparison donor triplets with and without heavy smokers (>10 pack years) and/or alcohol consumption (> 2 alcoholic beverages per day). The red line indicates the linear regression model (**adjusted r²**: 0.377; **Kendall’s tau**: 0.683). Only genes with a bimodal index < 1.3 were included in the analysis.


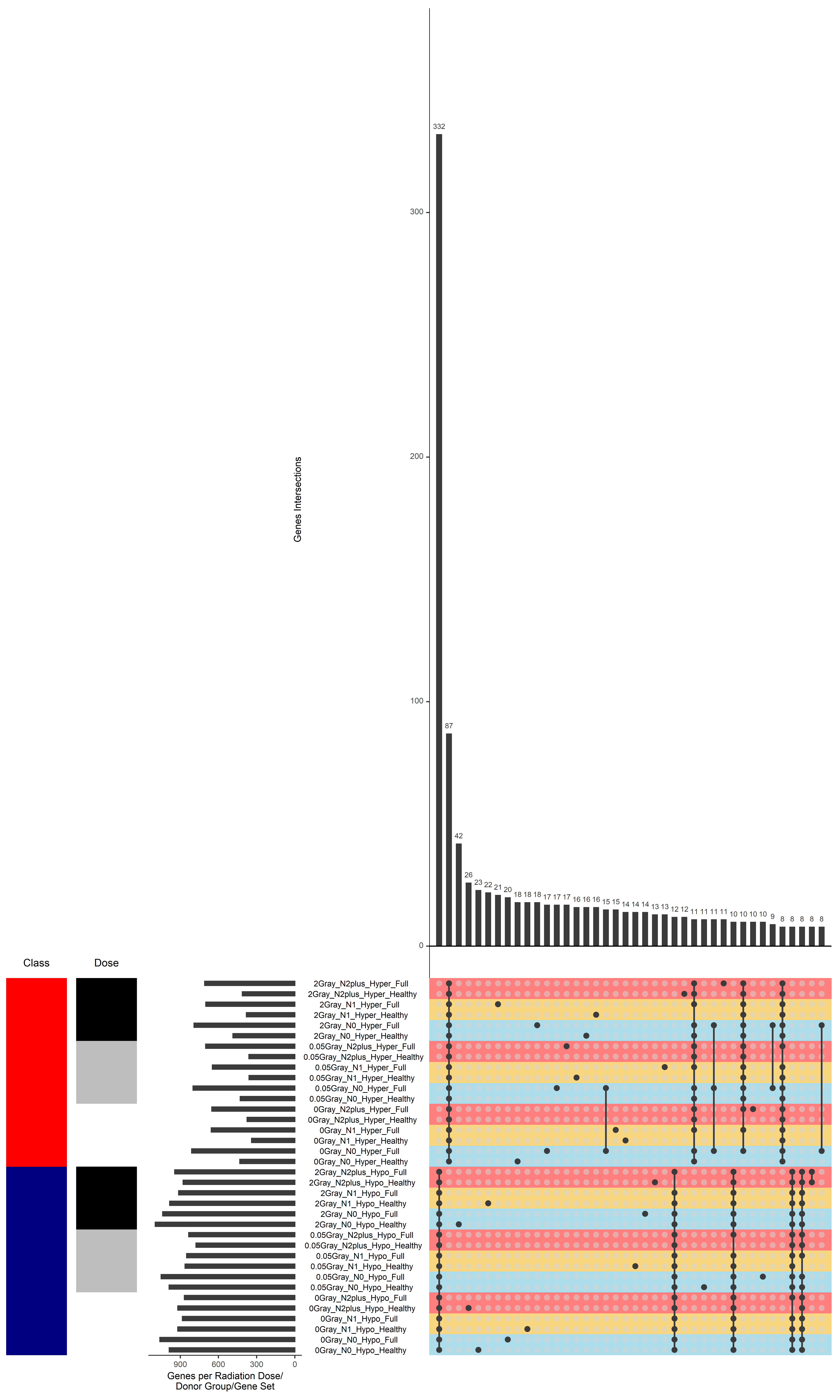
**Additional Files 7h:** Intersect graphs of overlapping gene classifications: Data are stratified by variability-classification, donor group (N0 = fibroblasts of cancer-free controls, N1 = fibroblasts of childhood cancer survivors without a second primary neoplasm, N2+ = fibroblasts of childhood cancer survivors with at least one second primary neoplasm), radiation dose, and additionally computed using only the 20 donor triplets (n=60) without heavy smokers (>10 pack years) and/or alcohol consumption (> 2 alcoholic beverages per day). Connected rows implicate that genes were identically classified in these data sets. Bars denote the summed number of identically classified genes among the vertically connected rows of data.


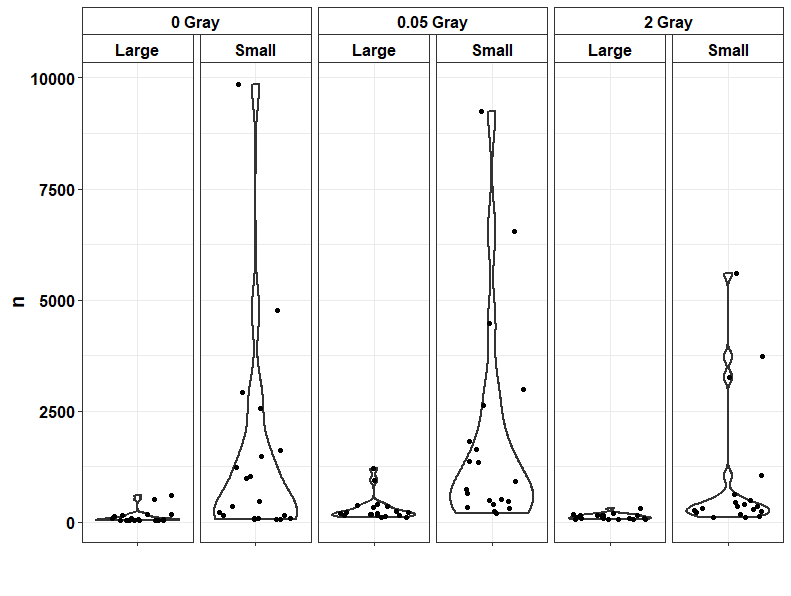


**Additional File 7i:** Density plots showing the number (n) of bimodal genes ( > 1.3) in 20 randomly assigned splits of the data in small (n=60) and large (n=96) splits, stratified by radiation dose. The small split can be seen to be more prone to largely higher numbers of bimodal genes. Regression found no other determinant than sample size in our meta data to explain this (data not shown).
